# Detection of queuosine and queuosine precursors in tRNAs by direct RNA sequencing

**DOI:** 10.1093/nar/gkad826

**Published:** 2023-10-09

**Authors:** Yu Sun, Michael Piechotta, Isabel Naarmann-de Vries, Christoph Dieterich, Ann E Ehrenhofer-Murray

**Affiliations:** Institut für Biologie, Lebenswissenschaftliche Fakultät, Humboldt-Universität zu Berlin, 10115 Berlin, Germany; Klaus Tschira Institute for Integrative Computational Cardiology, University Hospital Heidelberg, Heidelberg, Germany; Department of Internal Medicine III (Cardiology, Angiology, and Pneumology), University Hospital, Heidelberg, Germany; German Centre for Cardiovascular Research (DZHK)-Partner Site Heidelberg/Mannheim, Heidelberg, Germany; Klaus Tschira Institute for Integrative Computational Cardiology, University Hospital Heidelberg, Heidelberg, Germany; Department of Internal Medicine III (Cardiology, Angiology, and Pneumology), University Hospital, Heidelberg, Germany; German Centre for Cardiovascular Research (DZHK)-Partner Site Heidelberg/Mannheim, Heidelberg, Germany; Klaus Tschira Institute for Integrative Computational Cardiology, University Hospital Heidelberg, Heidelberg, Germany; Department of Internal Medicine III (Cardiology, Angiology, and Pneumology), University Hospital, Heidelberg, Germany; German Centre for Cardiovascular Research (DZHK)-Partner Site Heidelberg/Mannheim, Heidelberg, Germany; Institut für Biologie, Lebenswissenschaftliche Fakultät, Humboldt-Universität zu Berlin, 10115 Berlin, Germany

## Abstract

Queuosine (Q) is a complex tRNA modification found in bacteria and eukaryotes at position 34 of four tRNAs with a GUN anticodon, and it regulates the translational efficiency and fidelity of the respective codons that differ at the Wobble position. In bacteria, the biosynthesis of Q involves two precursors, preQ_0_ and preQ_1_, whereas eukaryotes directly obtain Q from bacterial sources. The study of queuosine has been challenging due to the limited availability of high-throughput methods for its detection and analysis. Here, we have employed direct RNA sequencing using nanopore technology to detect the modification of tRNAs with Q and Q precursors. These modifications were detected with high accuracy on synthetic tRNAs as well as on tRNAs extracted from *Schizosaccharomyces pombe* and *Escherichia coli* by comparing unmodified to modified tRNAs using the tool JACUSA2. Furthermore, we present an improved protocol for the alignment of raw sequence reads that gives high specificity and recall for tRNAs *ex cellulo* that, by nature, carry multiple modifications. Altogether, our results show that 7-deazaguanine-derivatives such as queuosine are readily detectable using direct RNA sequencing. This advancement opens up new possibilities for investigating these modifications in native tRNAs, furthering our understanding of their biological function.

## Graphical Abstract

**Graphical Abstract:**
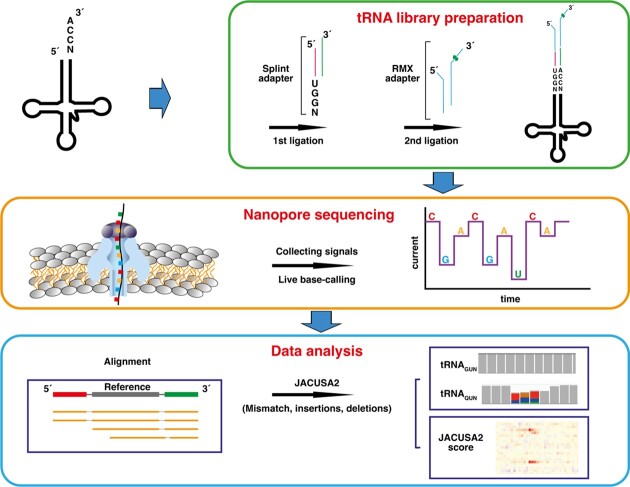

## Introduction

Transfer RNAs (tRNAs) carry many elaborate chemical modifications that affect tRNA folding, stability, codon recognition and maintenance of the mRNA reading frame and thus strongly contribute to the fidelity and efficiency of decoding (1–3). The greatest chemical diversity of tRNA modifications occurs in the anticodon loop, predominantly at positions 34 (the Wobble position) and 37, which are involved in the interaction with the mRNA during the process of codon-anticodon base pairing (4).

The importance of tRNA modifications for tRNA function necessitates adequate methods to reliably determine tRNA modifications in complex samples. While this has been an area of active development over the past few years, the currently available methods each have their own drawbacks. Such approaches include liquid chromatography coupled to mass spectrometry, immunoprecipitation with antibodies, as well as methods that rely on reverse transcription (RT) coupled to next-generation sequencing (NGS). For modifications that are RT-silent, prior chemical derivatization has been employed to create non-canonical nucleotides that increase misincorporation and/or RT-stops, thus generating a unique mutation signature. However, such methods do not directly detect the modification, and they cannot detect multiple modifications on the same (t)RNA strand (5–7).

An alternative approach is direct RNA sequencing using nanopores, as has been developed by Oxford Nanopore Technologies (ONT). This technology has enabled direct sequencing of full-length synthetic or native RNA molecules without reverse transcription or amplification steps, and has emerged as a promising method to detect modifications in RNA (and DNA) molecules (8,9). Briefly, the passage of single-stranded RNA in 3′ to 5′ direction through a nanopore, which is driven by voltage, results in characteristic changes in ionic current across the pore that depend on the chemical structure of the nucleobases. The modified nucleotides generally induce discernible shifts compared to canonical nucleotides, which allows further detection algorithms to recognize modifications. The ionic current signature is interpreted as individual bases using software from ONT (8). It should therefore, in principle, be possible to distinguish any nucleobase (modified or not) based on its unique chemical structure, which is expected to yield a specific current signature. This method thus is distinct from the above approaches in that it reads nucleotides directly along a single-stranded RNA without prior chemical modification or RT steps. So far, it has been employed to detect and map various modifications, such as *N^6^*-methyladenosine (m^6^A), 5-methyl-cytosine (m^5^C) and pseudouridine (Ψ) (10–13).

Here, we sought to harness direct tRNA sequencing to detect the modification of tRNAs with queuosine (Q) and Q precursors. Queuosine is a hypermodified 7-deaza-guanosine analog found at the Wobble position 34 in NAC/U-decoding tRNAs, namely tRNA^Asn^_GUU_, tRNA^Asp^_GUC_, tRNA^His^_GUG_ and tRNA^Tyr^_GUA_ (14,15). While Q is ubiquitous in most bacteria and eukaryotes (with the notable exceptions of *Saccharomyces cerevisiae* and *Arabidopsis thaliana*), only bacteria are capable of *de novo* Q biosynthesis (16,17). In bacteria, Q is derived from guanosine-5′-triphosphate (GTP), which is first converted to the intermediate 7-cyano-7-deazaguanine (preQ_0_) by consecutive enzymatic steps using GTP cyclohydrolase I, QueD, QueE and QueC (18,19). preQ_0_ is further reduced by the NADPH-dependent dehydrogenase QueF to the precursor base 7-aminomethyl-deazaguanine (preQ_1_), which is subsequently incorporated into the respective tRNAs (19,20). In this reaction, the bacterial tRNA-guanine transglycosylase (bTGT) exchanges the guanine 34 nucleobase for the precursor base preQ_1_ (21,22). While preQ_1_ is the preferred substrate for bTGT, it may also incorporate preQ_0_ into tRNAs if no preQ_1_ is available, as is the case in a *queFΔ* strain (20). The inserted preQ_1_ is subsequently modified to epoxyqueuosine (oQ) by the enzyme QueA, and oQ undergoes final reduction to Q by QueG (23,24).

Interestingly, in contrast to bacteria, eukaryotes depend on external sources like nutrients and/or microflora to acquire queuosine and its corresponding nucleobase queuine (q), because they lack the Q biosynthetic pathway (25). The salvaged queuine is directly installed at the Wobble position of the respective tRNAs by the eukaryotic TGT (eTGT), which, unlike the homodimeric bTGT, functions as a heterodimer comprised of queuine tRNA-ribosyltransferase catalytic subunit 1(QTRT1) and queuine tRNA-ribosyltransferase accessory subunit 2 (QTRT2) (26,27). Salvaged queuosine is hydrolyzed by the Qng1 enzyme to release queuine, the substrate of eTGT (28,29). In mammals, Q in the tRNA^Tyr^ and tRNA^Asp^ can be further glycosylated to galactosyl−Q (galQ) and mannosyl−Q (manQ), respectively (30–32). Interestingly, Q is involved in a modification circuit with 5-methyl-cytosine (m^5^C) in that Q modification of tRNA^Asp^ stimulates methylation of C38 by the Dnmt2 family of tRNA methyltransferases. While originally observed in *Schizosaccharomyces pombe*, this circuit has also been seen in *Dictyostelium*, mouse and human cells and thus may be a universal feature of Dnmt2 enzymes (33,34). Whether other tRNA modifications alter upon Q modification is not known.

As a modification at the Wobble position of tRNAs, the regulatory role of Q in translation has been investigated in eukaryotes. The Q-modified tRNAs with the GUN anticodon, in which G34 is modified to Q34, decode both NAC and NAU codons in the mRNA. The presence of Q equilibrates translational speed between the C- and U-ending codons, which is consistent with the hypothesis that modifications at the Wobble position compensate for lower stability of codon-anticodon interactions (35), though the effect can differ between organisms. In mouse and human cells, Q modification enhances the translational speed of U-ending codons (34). In the fission yeast *S. pombe*, Q modification enhances the translational speed of the C-ending codons for aspartate and histidine and decreases the speed of the U-ending codons for asparagine and tyrosine (36). In the protozoan parasite *Trypanosoma brucei*, Q-containing tRNAs preferentially decode the U-ending codons, and the availability of Q affects the translation of genes depending on their content of the U-ending codons (37,38). Furthermore, Q regulates translation accuracy in bacteria and *Schizosaccharomyces pombe* (36,39). On the organismal level, the absence of Q modification causes no major defects, but can reduce cell viability under stress conditions in some organisms (40). In mammalian cells, Q levels are enhanced upon arsenite exposure, which gears translation towards more efficient translation of proteins involved in energy metabolism (41). Manipulation of Q levels can furthermore aid mammalian cells fending off bacterial infection by sequestering Zn(II) that is a co-factor of Q biosynthetic enzymes (42). Moreover, the reduction of Q modification reduces the growth of cancer cells (43), thus making Q-modifying enzymes potential anti-cancer targets. Altogether, the medical relevance of Q reveals a need for methods to determine Q modification levels in tRNAs.

Traditional approaches to detecting and quantifying Q modification so far have not been sequencing-based and include liquid chromatography-mass spectrometry (LC/MS), or acryloylaminophenyl boronic acid (APB) gels combined with Northern blotting (44,45). However, both LC/MS and APB gels cannot detect Q at single-base resolution. More recently, with the development of next-generation sequencing (NGS), a method by coupling chemical treatment with RNA-seq has been established to investigate Q modification in single-base resolution, in which periodate-treated tRNAs yield deletions at the Q position in the sequencing data (46). Furthermore, in our earlier work, we labeled tRNAs *in vitro* and *in vivo* with a non-natural preQ_1_ derivative carrying an azide group, which, when combined with RNA-seq, can reveal the native substrates of TGT *in vivo* (47). Also, we recently developed a specialized reverse transcriptase/ polymerase that makes misincorporations opposite queuosine and pseudouridine and thus can be employed to detect Q modification by analyzing error profiles after NGS (48). However, as with earlier NGS-based approaches, these methods require chemical treatment and/or reverse transcription, which have the aforementioned disadvantages.

In this study, we have employed direct tRNA sequencing using ONT MinION to detect Q, preQ_1_ and preQ_0_ modification in tRNAs. To obtain end-to-end base calling, adapters were ligated to the 5′ and 3′ end of tRNAs that were either obtained by *in vitro* transcription, or were isolated from the yeast *S*. *pombe* and from *Escherichia coli*. Alignment of synthetic and biological as well as Q-modified and non-modified tRNAs revealed base miscalling, deletions and insertions at and around the Q position. The analysis of tRNAs from *E. coli* strains with defects in queuosine biosynthesis allowed us to distinguish between Q and its precursors preQ_1_ and preQ_0_. Furthermore, comparison of synthetic tRNAs with those obtained from *S. pombe* indicates that other tRNA modifications in principle can be detected with this method. Taken together, this study shows that direct RNA sequencing is a powerful tool to identify the modification of tRNAs with Q and its precursors at single-base resolution.

## Materials and methods

### *In vitro* synthesis of *S. pombe* tRNAs

*S. pombe* tRNAs (tRNA^Asp^, tRNA^Asn^, tRNA^Tyr^, and tRNA^His^) were *in vitro*-transcribed (IVT) from plasmids encoding the corresponding tRNAs using the TranscriptAid T7 High Yield Transcription Kit (Thermo Fisher Scientific). All plasmids used in this study are shown in Supplementary Table S1. Briefly, the sequence of the tRNAs including a T7 promotor and a ‘CCA’ tail was amplified from the plasmids pAE1688, pAE2240, pAE3371 and pAE3376 and cloned into the pJET1 vector with XhoI and ClaI. The oligonucleotides use for plasmid construction are shown in Supplementary Table S2. The resulting plasmids (pAE3633, pAE3634, pAE3635 and pAE3636) were linearized with NsiI, and 3 μg of the linear plasmid were used for *in vitro* transcription by T7 RNA polymerase according to the manufacturer's instructions. After a 7 h incubation at 37°C and subsequent DNase I treatment, the transcription reaction was purified using phenol/ chloroform/ isoamylalcohol extraction followed by gel filtration on Sephadex G50 (GE Healthcare).

### *In vitro* modification of tRNAs with queuosine

For *in vitro* queuosinylation of tRNAs, 10 μM of IVT tRNAs were incubated with 5 μM synthetic queuine (kindly provided by Hans-Dieter Gerber and Gerhard Klebe, Universität Marburg (49)) at 30°C for 7 h. The reaction mix also contained 200 nM human TGT (expressed and purified as described (28)), 50 mM Tris–HCl (pH 7.5), 20 mM NaCl, 5 mM MgCl_2_, and 2 mM dithiothreitol. Following incubation, tRNAs were purified using phenol-chloroform extraction and ethanol precipitation. 5′ phosphorylation was carried out following *in vitro* transcription and *in vitro* Q modification using T4 polynucleotide kinase (New England Biolabs) according to the manufacturer′s instructions.

### *S. pombe* growth and extraction of small RNAs

The *S. pombe* strain used in this study is AEP1 (*h^−^ leu1-32 ura4-D18 his3-D3*). Cells were grown in YES medium (5 g/l yeast extract, 30 g/l glucose, 250 mg/l adenine, 250 mg/l histidine, 250 mg/l leucine, 250 mg/l uracil, and 250 mg/l lysine) at 30°C and 140 rpm. This medium does not contain any queuine or queuosine. Synthetic queuine was added to the culture to a final concentration of 0.1 μM as appropriate.

To isolate small RNAs, *S. pombe* cells were cultured to an OD_600_ of 1, and 4 OD of cells were harvested by centrifugation. Small RNAs were extracted according to the manufacturer's protocol (PureLink™ miRNA Isolation Kit (Invitrogen)) with some adjustments. Briefly, cells were suspended in 1 ml Trizol (Ambion). The samples were vortexed for 2 min after adding 0.2 ml chloroform and glass beads, and then were centrifuged at 16 000 rcf, 4°C for 15 min. After adding 215 μl ethanol to the upper phase, the samples were transferred to a spin cartridge and centrifuged at 12 000 rcf for 1 min. 700 μl of ethanol was added to the flow-through, and the samples were transferred to a new spin cartridge followed by centrifugation at 12 000 rcf for 1 min. After washing the cartridge with wash buffer, small RNAs were eluted with 50 μl of DEPC-treated water. Small RNAs were deacylated for 30 min at 37°C in 100 mM Tris–HCl (pH 9).

### *E. coli* growth and extraction of small RNAs

The *E. coli* strains used in this study are shown in Supplementary Table S3. Except for *tgtΔ*, which was constructed in this study, the other strains were obtained from the Keio collection held at the Coli Genetic Stock Center (CGSC). The *tgtΔ* strain was constructed following the previously described strategy (50). Primers used for strain construction are listed in Supplementary Table S2.

The *E. coli* strains were grown in M9 medium (6.8 g/l Na_2_HPO_4_, 3 g/l KH_2_PO_4_, 0.5 g/l NaCl, 1 g/l NH_4_Cl, 2 mM MgSO_4_, 0.1 mM CaCl_2_, 0.4% glucose) at 37°C and 180 rpm. M9 medium for the deletion strains was supplemented with kanamycin (40 μg/ml). To isolate small RNAs, *E. coli* cells were grown to an OD_600_ of 1, and 4 OD of cells were harvested by centrifugation. The same method as described above for *S. pombe* was applied for the extraction of small RNAs from *E. coli*.

### Acryloyl aminophenylboronic acid (APB) gels and Northern blotting

To detect Q modification, 250 ng of IVT tRNAs or alternatively 300 ng of deacylated *S. pombe* small RNAs were mixed with an equal volume of 2 $ \times$ RNA loading buffer (New England Biolabs), denatured at 70°C for 5 min, and then separated on a 12% 7 M urea/TBE PAGE gel with 5 mg/ml 3-(acrylamido)-phenylboronic acid as previously described with a few modifications (44). Electrophoresis was performed in 1 $ \times$ TBE buffer for approx. 2 h at 30 mA. For IVT tRNA samples, the APB gel was stained after electrophoresis in a 1 $ \times$ TBE solution containing Sybr Gold nucleic acid gel stain (Thermo Fisher Scientific) for 10 min and then visualized using a Gel Doc™ XR^+^ system (Bio-Rad). For Northern blotting, the electrophoresed gel was transferred onto a Biodyne B Nylon membrane (0.45 μm) in 1 $ \times$ TAE at 150 mA, 4°C for 90 min. tRNA^Asp^ was detected using the Chemiluminescent Nucleic Acid Detection Module Kit (Thermo Fisher Scientific), and DIG Easy Hyp buffer (Roche) was used for the first blocking. Hybridization was performed at 60°C overnight using a denatured biotinylated probe (Supplementary Table S2) with a final concentration of 30 ng/ml. The blot was imaged using the ChemiDoc Imaging system (Bio-Rad).

### Library preparation for Nanopore sequencing

The preparation of tRNA libraries was carried out using the SQK-RNA002 kit (Oxford Nanopore Technologies) following the method previously described (51). Briefly, 20 pmol of IVT tRNA^Asp^ were used as input material. To promote ligation of sequencing adapters to tRNA, 16 pmol of custom double-stranded splint adapter (with CGGU overhang complimentary to 3′ termini of tRNA^Asp^ GCCA (51), Supplementary Table S2) were used for the first ligation. The first ligation reaction was performed in a DNA LoBind tube at 25°C for 45 min. It also contained 1 $ \times$ RNA ligase2 buffer (New England Biolabs), 5% PEG 8000, 2 mM ATP, 6.25 mM MgCl_2_, 6.25 mM DTT and 0.5 units/μl T4 RNA ligase 2 (10 000 units/ml) in a total volume of 20 μl. For library preparation of mixtures of IVT tRNAs, 10 pmol of each of the four tRNAs (tRNA^Asp^, tRNA^Asn^, tRNA^Tyr^ and tRNA^His^) and 16 pmol of each of the two splint adapters (splint adapters with CGGU and UGGU overhang) were used in a reaction volume of 40 μl. Library preparation for tRNAs isolated from cells was performed using 40 pmol of deacylated small RNAs and 32 pmol of splint adapter (8 pmol of each of the four splint adapters). Ligation reaction samples were subsequently electrophoresed on a 7 M urea/TBE PAGE gel (8%), and ligation products were excised and purified using the ‘crush and soak’ method (52). The concentration of gel-purified ligation products was measured with the Qubit RNA HS Assay Kit (Invitrogen). The second ligation of splint-ligated tRNAs to the RNA Adapter (RMX, included in the SQK-RNA002 kit) and subsequent library purification were performed following the Nanopore Sequence-Specific Direct RNA sequencing protocol, except that incubation time of the second ligation was increased from 10 to 30 min to improve ligation efficiency.

### Nanopore sequencing

Samples were sequenced on a MinION Mk1C device using MinION FLO-MIN106D R9.4.1 flow cells (Oxford Nanopore Technologies). After priming the flow cell, 20 μl of prepared sequencing library mixed with 17.5 μl of nuclease-free water and 37.5 μl of RNA Running Buffer (RRB, included in the SQK-RNA002 kit) was loaded onto the flow cell. Sequencing runs were controlled by the MinKNOW software (Oxford Nanopore Technologies, version 22.10.5). Sequencing was typically carried out for 12–48 h depending on the number of active pores.

### Data processing

#### Reference curation

As reference sequences for *S. pombe* tRNAs, all unique mature tRNAs from GtRNAdb and all unique mitochondrial tRNAs from Ensembl Fungi were combined (total: 86 sequences), and the 5′ and 3′ splint adaptor sequences were added. For *E. coli*, sequences of all unique mature tRNAs were taken from GtRNAdb, and splint adaptor sequences were added as above.

#### Base calling and alignment

Base calling was done with Guppy version 6.3.8 (available to Oxford Nanopore Technologies customers via their community, https://nanoporetech.com/community) in high-accuracy mode. The resulting fastq reads were processed to convert Us to Ts. Both the reads that were called as ‘passed’ and ‘failed’ were employed, since we reasoned that tRNAs with many modifications may hence be called as ‘failed’, even though they constitute true tRNA reads. Sequence alignment was performed on ‘passed’ and ‘failed’ fastq files separately using an optimal local sequence alignment approach (Smith-Waterman) as implemented in parasail (53) against the reference database. Alignments are ranked according to their score. Thus, alignments of the same score have the same rank and multiple alignments with rank 1 will be retained. The traditional method for assigning statistical significance of these alignments is by simulation (54). In our work, we simply reversed input sequences to produce random alignments. This approach preserves the nucleotide composition and sequence length, which are the only relevant determinants of an alignment score. The respective alignment score distributions for real and random alignments are used to define an alignment score cutoff at a level of 95% specificity (true negatives/(true negatives + false positives) = 0.95). Additional details and a comparison to current heuristic i.e. suboptimal approaches can be found in Supplementary Materials and Methods. The respective source code to compute alignments and assess their significance is available via https://github.com/dieterich-lab/QutRNA. BAM files from ‘passed’ and ‘failed’ reads were then merged and visualized using Integrated Genome Viewer (IGV, version 2.15.2).

#### Analysis of base calling errors

For error calling, BAM files from ‘passed’ and ‘failed’ reads were merged and processed using JACUSA2 (version 2.0.4, (55)). Scores were processed and visualized using R scripts. Briefly, results from JACUSA2 were parsed to calculate the Mis + Del + Ins score, which corresponds to the sum of JACUSA2 mismatch, insertion and deletion scores. Furthermore, mismatch, insertion and deletion rates were computed from JACUSA2 results. Resulting scores were processed and visualized in R. The entire end-to-end workflow is available at https://doi.org/10.5281/zenodo.8325692.

## Results

### Detection of queuosinylation on *in vitro*-transcribed tRNAs

In this study, we sought to harness direct RNA sequencing to detect Q modification in tRNA (Supplementary Figure S1). For this purpose, we adapted the method developed by Thomas *et al.* (51). Briefly, tRNA molecules were ligated to double-stranded splint adapters that have an RNA segment, which brings RNA sequences immediately adjacent to the ligated tRNA and improves reading by the ONT base calling software. Ligation products were purified from a denaturing PAGE gel, and the excised and purified material was ligated to the ONT motor-associated sequencing adapter. Ionic current traces were interpreted using the Guppy software, and the resulting sequence reads were aligned to the reference sequence using parasail (see Materials and Methods for details) (53). Subsequently, JACUSA2 (55) was employed to detect base miscalling, deletions and insertions, and JACUSA2 scores were assigned to each tRNA position based on the comparison between tRNAs with and without Q modification (+Q/−Q). High JACUSA2 scores are indicative of a modification at the respective position.

**Figure 1.**
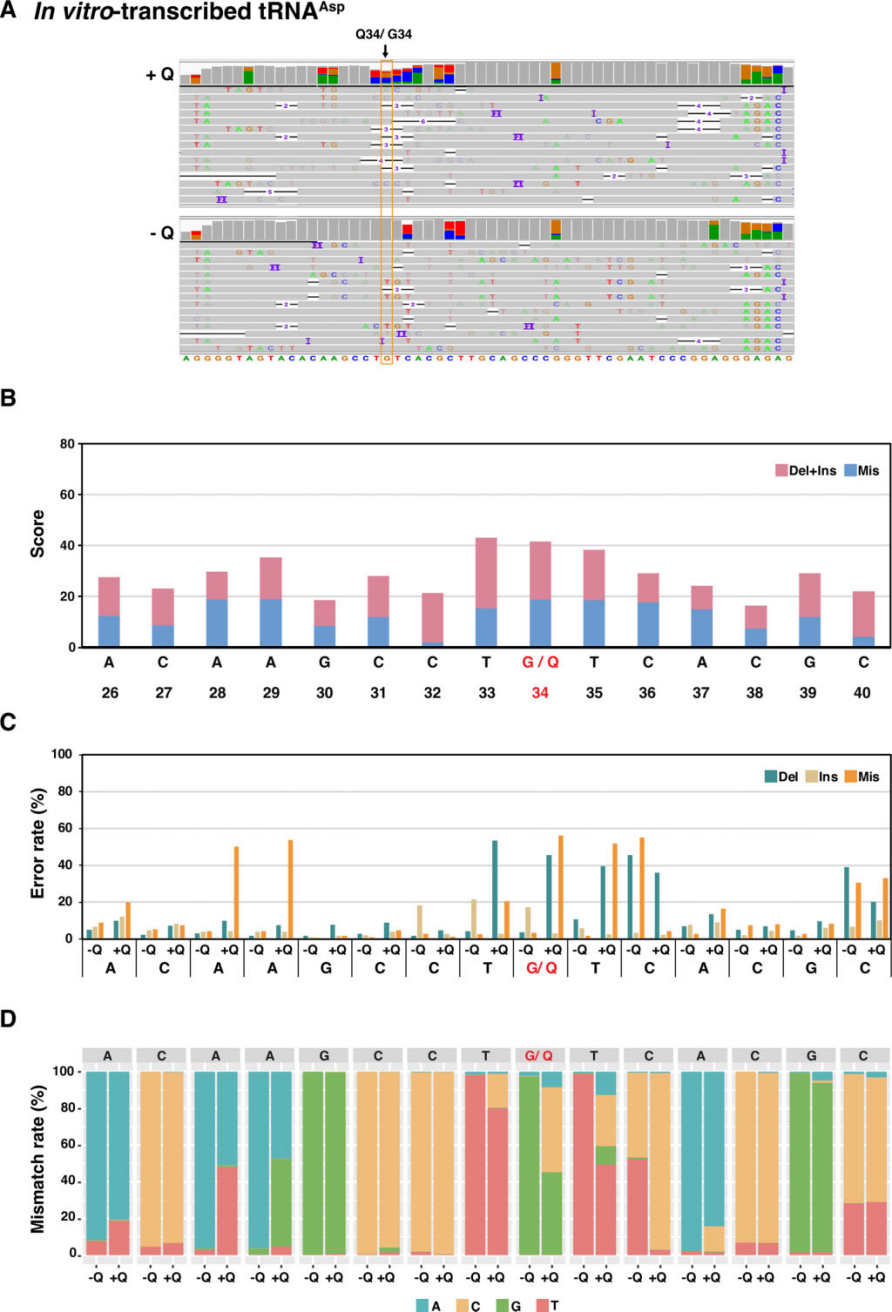
Detection of Q modification on *in vitro*-transcribed tRNA^Asp^ by nanopore direct RNA sequencing. (**A**) IGV snapshots of *in vitro*-transcribed tRNA^Asp^ with (+Q) or without Q-modification (−Q). The Q position is indicated above the coverage plot. Gray bars in the coverage plots represent positions with a misincorporation rate of less than 30%. The positions where misincorporation rates are equal or more than 30% is shown in color. (**B**) JACUSA2 scores of Q and the surrounding nucleotides in tRNA^Asp^. The scores are derived from JACUSA2 call-2 analysis of *in vitro*-transcribed tRNA^Asp^ ± Q, considering deletions and insertions (pink) as well as mismatches (blue). (**C)** Comparison of error rates (deletions, insertions and mismatch rates) of *in vitro*-transcribed tRNA^Asp^ ± Q on the same sites as in (B). The error rates were calculated by JACUSA2. (**D**) Comparison of mismatch signature from *in vitro*-transcribed tRNA^Asp^ ± Q. At each position, the frequencies of individual bases as given by JACUSA2 are shown.

We first sought to establish the method using *in vitro*-transcribed tRNA^Asp^ that was either unmodified or Q-modified *in vitro* using hTGT. The modification level after treatment with hTGT was >98%, as judged by APB gel electrophoresis (Supplementary Figure S2A). The resulting aligned reads were visualized using the integrated genome viewer (IGV) software, where grey bars in the coverage plot indicate positions with error rates of <30% (Figure 1A). Importantly, this showed increased errors as well as short deletions at and around the Q34 position of tRNA^Asp^. There was also base miscalling at some sites in the non-modified tRNA^Asp^, which may be a result of the sequence context or of the secondary structure of the tRNA (Figure 1A).

Next, JACUSA2 was employed on Q- versus non-modified tRNA^Asp^, which assigns a score to each position based on the comparison. This analysis assigned a high score to the Q34 site, as well as to positions 5′ and 3′ of Q34 (Figure 1B). A closer inspection of the error profiles showed that Q34 caused increased levels of deletions and mismatches compared to G34. Furthermore, U35 also displayed increased mismatches and deletions. Since direct sequencing proceeds from 3′ to 5′, this indicates that U35 entering the nanopore already senses Q34. Also, we observed a ‘trail’ of higher error rates 5′ to Q34, i.e. as Q34 is passing through and exiting the nanopore (Figure 1C). The miscalling at Q34 was predominantly C, and U33 and U35 were also predominantly miscalled as C (Figure 1D). Curiously, there was relatively high error calling at A28 and A29, and A28 was mainly miscalled as U, whereas A29 was mainly miscalled as G (Figure 1D), even though neither site is modified on the *in vitro* transcript. Altogether, these results showed that Q34 can unequivocally be distinguished from G by the comparison of error profiles between non- and Q-modified template.

We next investigated the detection of Q modification in the other three Q-tRNAs, tRNA^Asn^, tRNA^His^ and tRNA^Tyr^. For this, all four Q-tRNAs were individually *in vitro*-transcribed and Q-modified as described above, and an equimolar mixture of the respective unmodified or modified transcripts were adapter-ligated and subjected to direct RNA sequencing. Because the tRNAs have different bases immediately 5′ to the CCA tail, this entailed ligation with a mixture of splint adapters. As for tRNA^Asp^, the other three Q-tRNAs showed higher JACUSA2 scores and increased base miscalling and deletions at and around Q34, though the patterns differed somewhat between the different templates. In tRNA^Asn^ and tRNA^His^, base miscalling was seen in a broader segment compared to tRNA^Asp^, and extended from positions 28 to 36 in tRNA^Asn^ and 30 to 37 in tRNA^His^ (Supplementary Figures S3, S4 and S5). In tRNA^Tyr^, higher error rates were seen at positions 29 to 35 (Supplementary Figure S6). However, the UQU sequence showed a similar misincorporation profile in all Q-tRNAs, with both Us and Q34 frequently miscalled as C (Supplementary Figures S3–S6). Taken together, this showed that Q modification was detectable by direct RNA sequencing in all four known Q-modified tRNAs by comparing nanopore sequencing errors of tRNAs with or without Q modification using JACUSA2. The miscalling profile caused by Q modification varied depending on the sequence context in the tRNA.

### Nanopore sequencing of tRNAs from *S. pombe*

We next applied this sequencing method to total tRNA purified from *S. pombe*. In this yeast, Q modification levels can easily be controlled by queuine levels in the growth medium, because it lacks the Q-biosynthetic enzymes and therefore fully relies on externally provided queuine for tRNA modification. *S. pombe* has a TGT enzyme that consists of the two subunits Qtr1 and Qtr2, the homologs of QTRT1 and QTRT2 in higher eukaryotes, which exchanges G34 for Q in the four known Q-tRNAs. Q modification of tRNA^Asp^ stimulates Dnmt2-dependent methylation of C38 to m^5^C (33).

**Figure 2.**
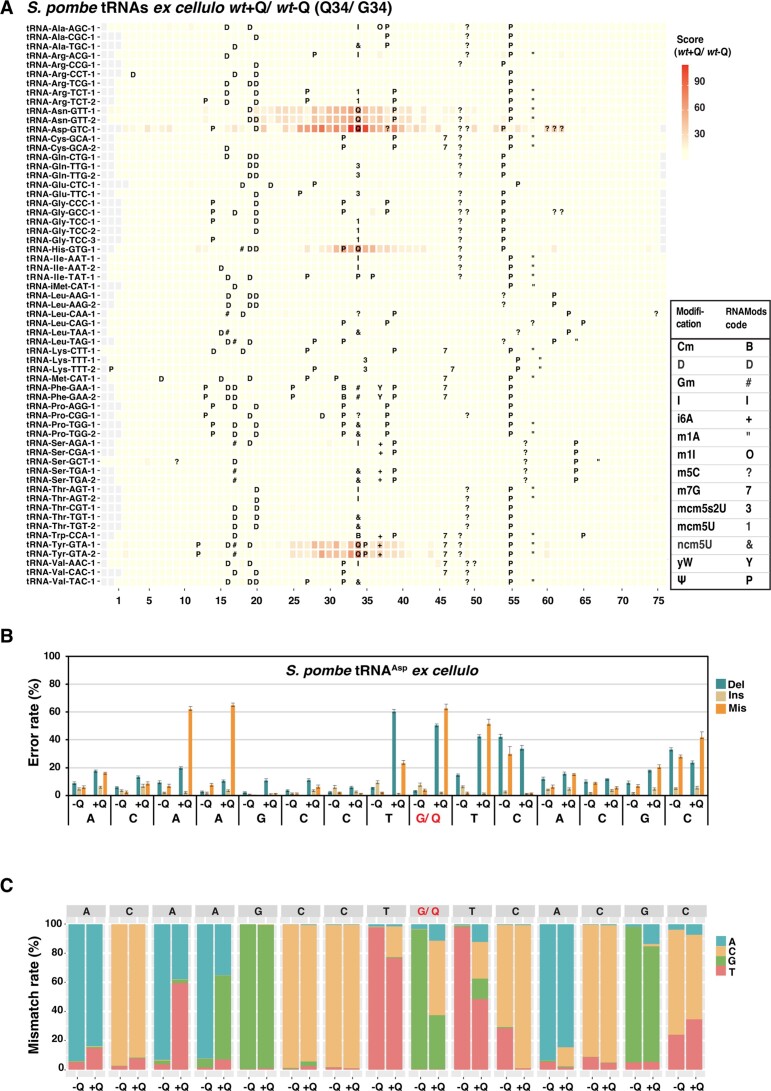
Detection of Q modification on *S. pombe* Q-tRNAs by direct RNA sequencing. (**A**) JACUSA2 scores of tRNAs (‘*ex cellulo*’) from wild-type *S. pombe* cells cultivated in the absence (*wt*− Q) or presence (*wt*+ Q) of queuine. The position of each tRNA is aligned by the anticodon, the first nucleotide of which is position 34. Known and predicted modification sites (Supplementary Dataset S1) are marked using the unified RNAMods code for modified residues as used in the Modomics database (67), and the bottom-right table provides information for each modification and its corresponding code. The JACUSA2 scores are the aggregate of three replicates per condition. (**B**) Comparison of error rates of tRNA^Asp^ from *S. pombe wt* cells cultivated in the absence (−Q) and presence (+Q) of queuine. The values are the average of three biological replicates per condition, and the error bars represent standard deviations. (**C**) Misincorporation signature of tRNA^Asp^ from *S. pombe wt* cells cultivated in the absence (−Q) or presence (+Q) of queuine.

For direct sequencing, total tRNA was isolated from wild-type (*wt*) *S. pombe* cells grown in the presence (*wt* + Q) or absence (*wt* − Q) of queuine. Under these conditions, the Q-tRNAs are either fully Q-modified, or have no Q modification (Supplementary Figure S2B). For clarity, we call such tRNAs ‘*ex cellulo*’ here in order to distinguish them from *in vitro*-transcribed tRNAs. The tRNAs were ligated with the mixture of four double-stranded splint adapters to capture all possible tRNAs. Ligated tRNAs were gel-purified as above, ligated to the ONT sequencing adapters and subjected to direct sequencing. The experiment was conducted with three biological samples per condition, and the read signatures and scores of aligned reads comparing *wt* + Q to *wt* − Q were determined by JACUSA2. The scores were plotted for each tRNA and overlayed with the known or predicted positions of Q and other modifications in order to obtain a comprehensive view of the modification map (Figure 2A and Supplementary Dataset S1). This showed a high score at and around the known Q-modified positions, spanning a varying number of bases both 5′ and 3′ of Q34, much like what was found in *in vitro*-transcribed tRNA. Of note, the read signature of G34/ Q34 and its adjacent positions in Q-tRNAs *ex cellulo* was similar to that of *in vitro*-transcribed samples (Figure 2B, and Supplementary Figures S7–S9). For instance, in tRNA^Asp^ from *wt* + Q, the positions U33, Q34 and U35 showed higher deletion and misincorporation rates compared to the same positions in tRNA^Asp^ from *wt*−Q, and the miscalling frequently was to a C (Figure 2B and C). Also, the A28 and A29, which showed unusual signatures in *in vitro*-transcribed tRNAs despite not being modified, showed high mismatch rates and also were frequently miscalled to U and G, respectively, like in the *in vitro* tRNAs (Figure 2C). Of note, this analysis showed that the three biological replicates of each condition give similar error profiles, showing that the methodology is highly reproducible. Overall, these results demonstrated that direct RNA sequencing allows the detection of Q modification on Q-tRNAs *ex cellulo* from *S. pombe*, i.e. on tRNAs that carry several other tRNA modifications. The Q positions could be assigned by comparing read features from Q-modified and non-modified tRNAs using JACUSA2.

A next interesting question was whether positions other than Q34 show high JACUSA2 scores, which would be indicative of changes at other modification positions in the presence or absence of Q modification. In our earlier work, we have shown that the level of m^5^C at C38 of tRNA^Asp^ is controlled by Q modification levels in that C38 is methylated to 15% in *wt* − Q and to > 99% in *wt* + Q (33). The JACUSA2 score at this position was relatively high (Figure 2A), which could be interpreted as an increase in methylation. However, this site is also ‘hidden’ under the broad pattern distribution of higher scores around Q34, such that it is difficult to distinguish this effect from m^5^C itself. Furthermore, mildly increased scores were observed at positions 60, 61 and 62 of tRNA^Asp^, and these sites are known to carry m^5^C (56). However, our earlier analysis has shown that the methylation level at these sites remains unaltered in +Q and −Q conditions, such that the reason for the higher score remains to be determined. Altogether, the modification map of tRNAs *ex cellulo* in *S. pombe* suggests that no modification sites other than Q34 are altered in +Q compared to −Q conditions. There are two caveats to this interpretation: (i) sites close to Q34 cannot be evaluated due to the ‘trail’ of JACUSA2 scores around Q34 and (ii) the changes in ionic current as the nucleobases pass through the nanopore are interpreted by proprietary software by ONT, which may not be able to distinguish some modifications.

### Identification of other modification sites in Q-tRNAs from *S. pombe*

**Figure 3.**
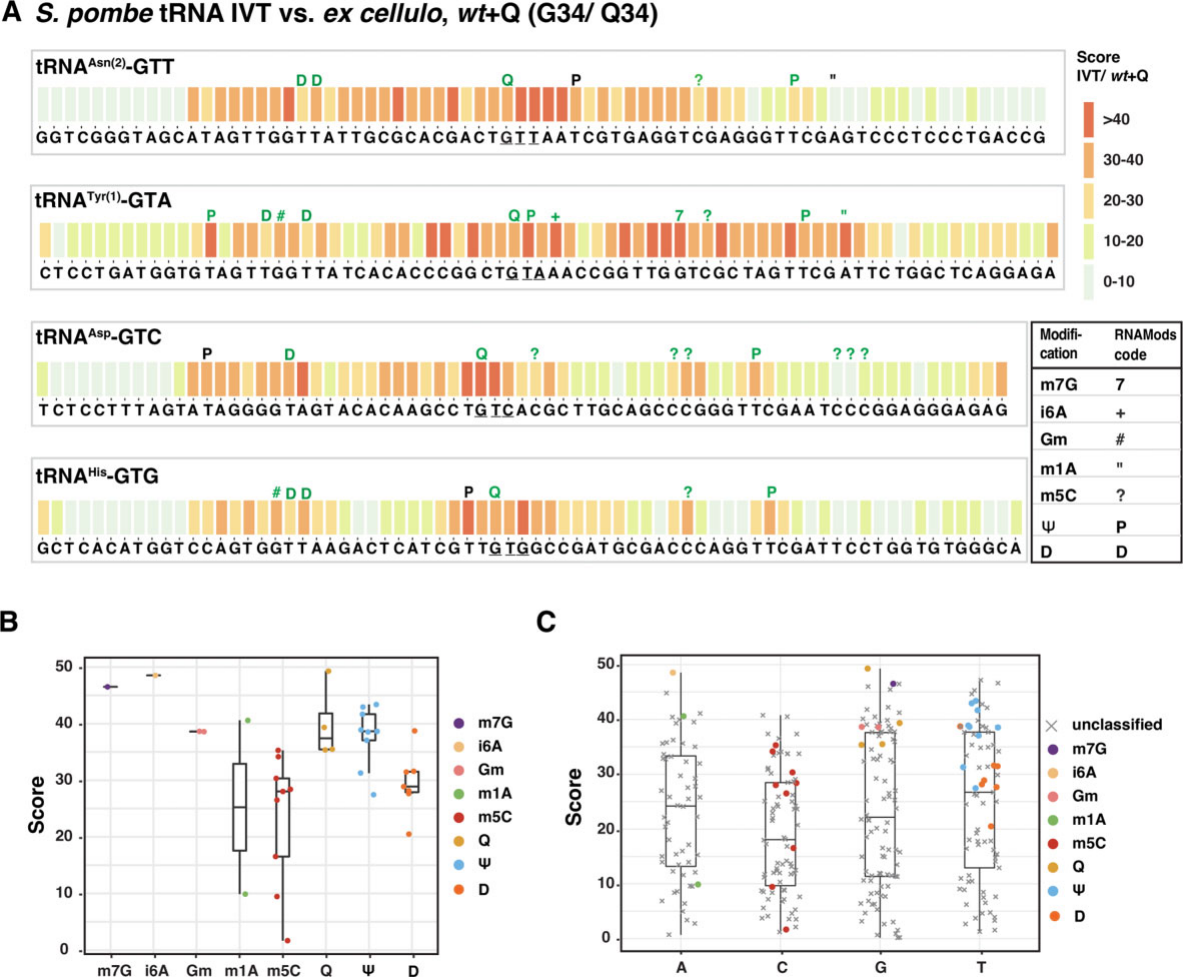
Detection of tRNA modifications in *S. pombe* by comparison of nanopore sequencing errors in tRNAs *ex cellulo* to *in vitro*-transcribed tRNAs. (**A**) JACUSA2 score of comparison between *in vitro*-transcribed (IVT) tRNAs and the corresponding tRNAs from *S. pombe wt* cells cultivated in the presence of queuine (*wt*+ Q). Known (green) and predicted (black) modification sites are indicated above the columns. (**B**) JACUSA2 score signatures of modifications present in Q-tRNAs. The modifications analysed are m^7^G (*n* = 1), i^6^A (*n* = 1), Gm (*n* = 2), m^1^A (*n* = 2), m^5^C (*n* = 9), Q (*n* = 4), Ψ (*n* = 9) and D (*n* = 7). Each dot represents an individual data value. (**C**) Distribution of JACUSA2 scores for each nucleobase. The coloured dots indicate the values for sites with known or predicted modifications as in (**B**), other sites are shown in grey.

In a next step, we sought to explore whether modifications other than Q can be detected by direct RNA sequencing. This was possible for the four Q-tRNAs, since we had obtained sequencing datasets from *in vitro*-transcribed tRNAs, which by nature are nonmodified at all sites, and tRNAs *ex cellulo*, which on average carry 13 tRNA modifications (4). For this purpose, the JACUSA2 scores were computed for *in vitro*-transcribed Q-tRNAs (not Q-modified) versus *ex cellulo* Q-tRNAs (*wt* + Q) and plotted alongside known or predicted sites of modification (Figure 3A). High scores were obtained at many positions along the four Q-tRNAs, which might be the result of a modification at such sites. However, it is also possible that some modifications leave a ‘trail’ of base miscallings, much like for Q, such that true modifications may be difficult to distinguish. A compilation of the scores at the modification sites showed high scores for m^7^G, i^6^A, Gm, Q, Ψ and D (Figure 3B). For m^5^C, some positions gave intermediate scores (ca. 25–35), whereas three sites gave lower scores (below 17). Closer inspection revealed that the lower-scoring sites are those that have lower levels of m^5^C modification (tRNA^Asp^ C60, 61 and 62, around 20% to 35%, (56)). For m^1^A, position 58 of tRNA^Tyr^ had a high score, whereas the score of position 58 of tRNA^Asn^ was low. Given that m^1^A58 in tRNA^Tyr^ has been demonstrated (57), whereas m^1^A58 modification on tRNA^Asn^ was inferred from *S. cerevisiae*, this suggests that position 58 carries m^1^A in tRNA^Tyr^, but not tRNA^Asn^, in *S. pombe*.

It was also of interest to compare the scores of the known modified sites of a particular base to that of other positions. These are either unmodified, or the modification is not yet known at this site in *S. pombe*. This analysis showed that there was a trend in the sense that known modified sites showed higher scores (Figure 3C). It therefore can be postulated that at least some of the high-scoring sites indeed are modified, though possible error pattern ‘trails’ must be taken into consideration.

We looked more specifically for whether m^5^C38 can be detected by determining the JACUSA2 score of *in vitro*-transcribed tRNAs that were Q-modified *in vitro* to tRNAs *ex cellulo* from *wt* + Q. The former has only Q, but no m^5^C38, on tRNA^Asp^, whereas the latter carries both Q and m^5^C38. Here, the score at C38 of tRNA^Asp^ was low (Supplementary Figure S10A), indicating that m^5^C cannot be detected at this position, possibly because the site is too close to Q34.

Two other sites that were of interest are Ψ35 and i^6^A37 on tRNA^Tyr^. Both showed high scores in tRNAs *ex cellulo* from *S. pombe* (+Q/−Q, Figure 2A), which could either come from the ‘Q trail’, or could suggest that the level of the two modifications is regulated by Q modification. However, scores for non-modified *in vitro* tRNA transcripts to *ex cellulo* tRNAs from wt−Q (i.e. both not Q-modified) showed a high score at U35 and A37 (Supplementary Figure S10B). These results showed that Ψ35 and i^6^A37 existed in tRNA^Tyr^ with Q34 or G34, indicating that Q modification does not regulate these modifications.

Taken together, this analysis indicated that the modifications m^7^G, i^6^A, Gm, m^1^A, Ψ and D are detectable by direct RNA sequencing and comparing non-modified tRNAs to tRNAs isolated from cells.

### Detection of Q, preQ_1_ and preQ_0_ in tRNAs from *E. coli*

The production of Q-modified tRNAs in bacteria proceeds through multiple biosynthetic steps, with intermediates containing, among others, preQ_0_ and preQ_1_. It therefore was of interest to see whether these Q precursors result in similar base miscalling as Q in direct RNA sequencing. The respective tRNAs can be obtained from *E. coli* strains lacking the respective biosynthetic enzymes. preQ_1_ (7-aminomethyl-7-deazaguanine)-containing tRNAs are present in *queAΔ* bacteria, because QueA converts preQ_1_ on tRNAs to epoxyqueuosine (oQ) (58). *queF* encodes the preQ_0_ reductase that converts preQ_0_ (7-cyano-7-deazaguanosine) to preQ_1_. In the absence of preQ_1_ in *queFΔ* strains, the bacterial tRNA guanine transglycosylase TGT can also incorporate preQ_0_ into tRNAs, though the extent of preQ_0_ modification in such strains is not known (20,59). Additionally, we sequenced tRNAs from *tgtΔ* as a control, since the respective Q-tRNAs carry G34.

**Figure 4.**
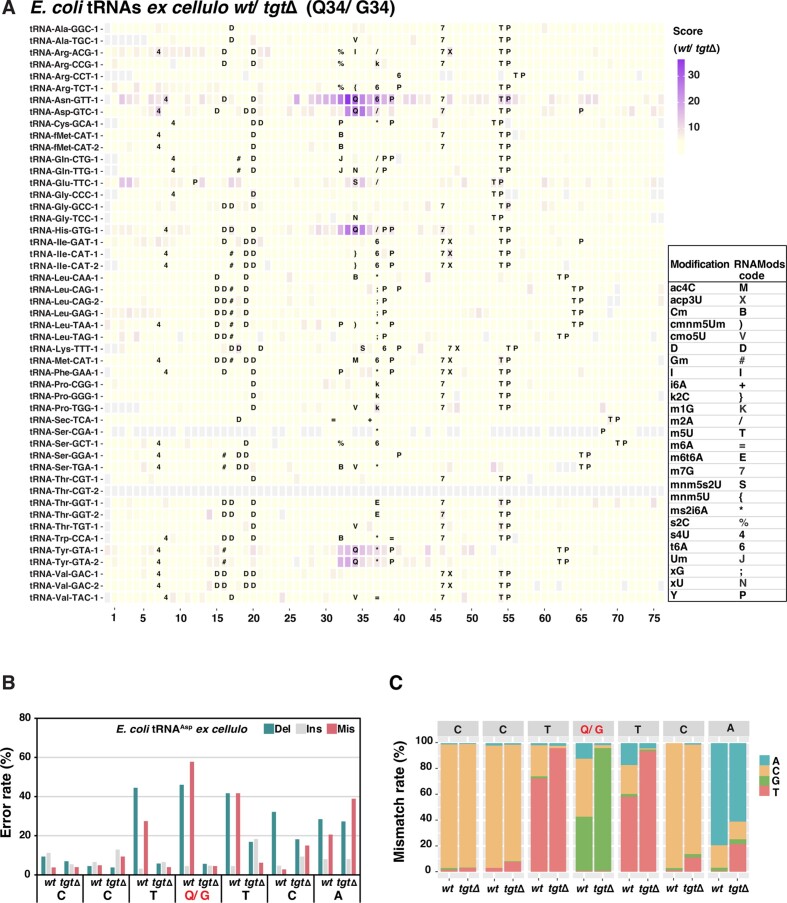
Detection of Q in tRNAs from *E. coli* by direct RNA sequencing. (**A**) JACUSA2 scores of total tRNA from *E. coli* (*ex cellulo*) *wt* versus *tgtΔ*. The position of each tRNA is aligned by the anticodon, the first base of which is at position 34 in the alignment. Known modifications at each position of the tRNAs is indicated using the RNAMods code (bottom-right table). (**B**) Comparative analysis of error rates of tRNA^Asp^ from *E. coli wt* versus *tgtΔ*. (**C**) Mismatch features of tRNA^Asp^ from *E. coli wt* and *tgtΔ*.

To investigate the detection of Q and Q precursors in tRNA, total tRNA from a wild-type (*wt*) and the respective Q biosynthesis mutants was isolated and subjected to direct RNA sequencing as described above for *S. pombe*. JACUSA2 scores of the comparison of *wt* and *tgtΔ* showed high scores at and around the Q34 positions of the four Q-tRNAs (Figure 4A), reinforcing the notion that Q modification is readily detectable by direct RNA sequencing in *E. coli*, as is the case in *S. pombe*. However, Q34 in *E. coli* tRNA^Asp^ resulted in a narrower error profile compared to that in *S. pombe* tRNA^Asp^ (Figure 4B), a feature that was also seen to tRNA^His^ and tRNA^Tyr^, but not tRNA^Asn^ (Supplementary Figures S11 and S12). Also, Q modification was mainly misread as C in *E. coli* tRNAs, as was the case in samples from *S. pombe* (Figure 4C). As above for *S. pombe*, it is difficult to conclude whether any modification sites other than position 34 are affected by the absence of TGT, especially for sites close to Q. Possibly, m^5^U54 and Ψ55 of tRNA^Asn^ are reduced in *tgtΔ*. Also, the JACUSA2 scores for tRNA^Thr^ (CGT-2) were absent along its whole length (Figure 4A), which we attribute to low coverage of this tRNA.

**Figure 5.**
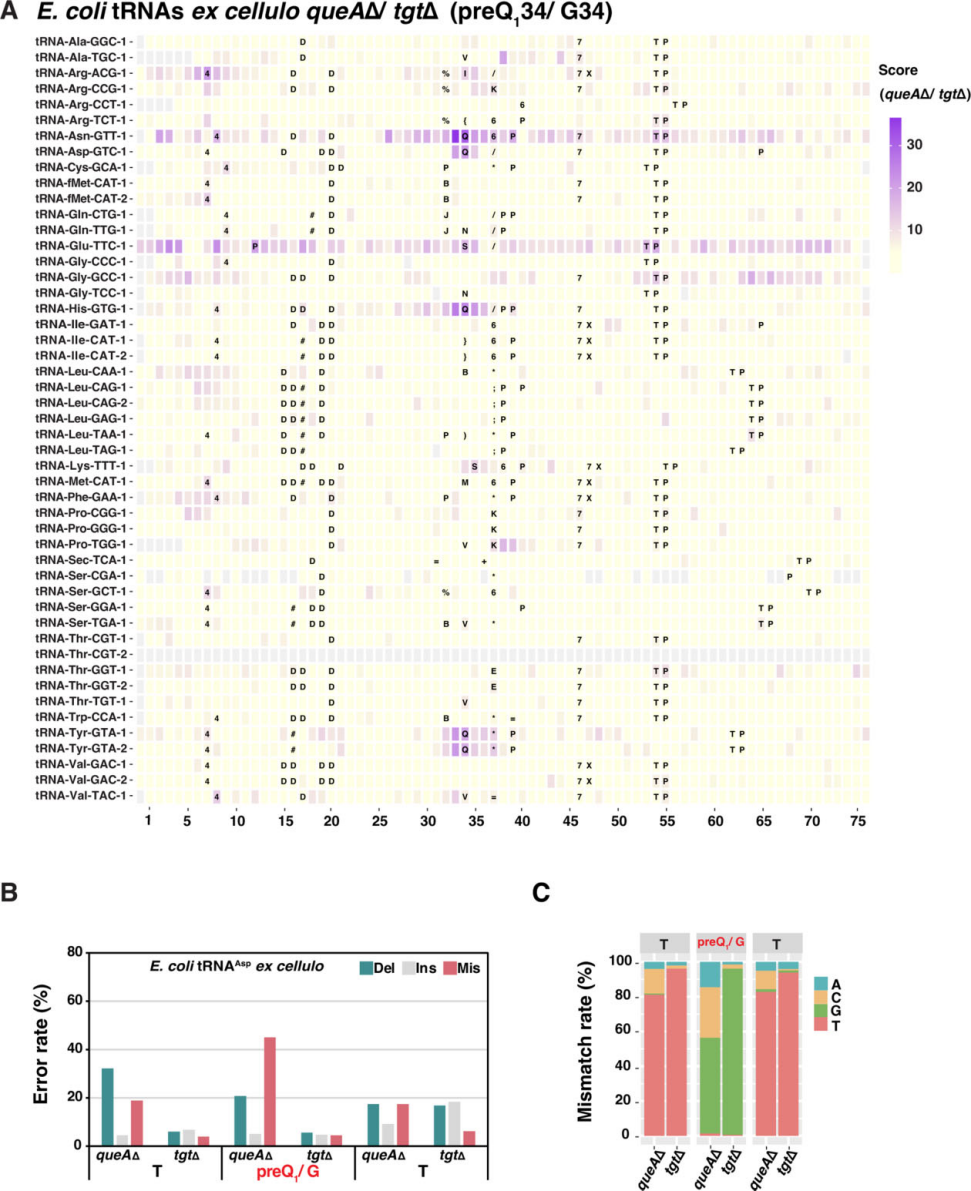
Detection of preQ_1_ in tRNAs from *E. coli*. (**A**) JACUSA2 scores of total tRNA from *E. coli queAΔ* versus *tgtΔ*. The position of each tRNA is aligned by anticodon, the first base of which is positioned 34. Representation as in Figure 4A. (**B**) Comparative analysis of error rates of tRNA^Asp^ from *E. coli queAΔ* versus *tgtΔ*. (**C**) Mismatch features of tRNA^Asp^ from *E. coli queAΔ* and *tgtΔ*.

We next sought to detect preQ_1_ by direct RNA sequencing. For this purpose, aligned reads from *queAΔ* and *tgtΔ* were compared to each other by JACUSA2. Indeed, high scores were observed in the four known Q-tRNAs at the preQ_1_-modified site and in the neighborhood (Figure 5A). Compared to Q modification on tRNA^Asp^, preQ_1_ lead to lower mismatch and deletion rates (Figure 5B, and Supplementary Figures S13 and S14). As for Q, preQ_1_ was mostly misread as C (Figure 5C, and Supplementary Figures S13 and S14). Collectively, these results indicate that preQ_1_, like Q, can be detected by direct RNA sequencing, and that the features of base miscalling for preQ_1_ differ from those of Q modification.

**Figure 6.**
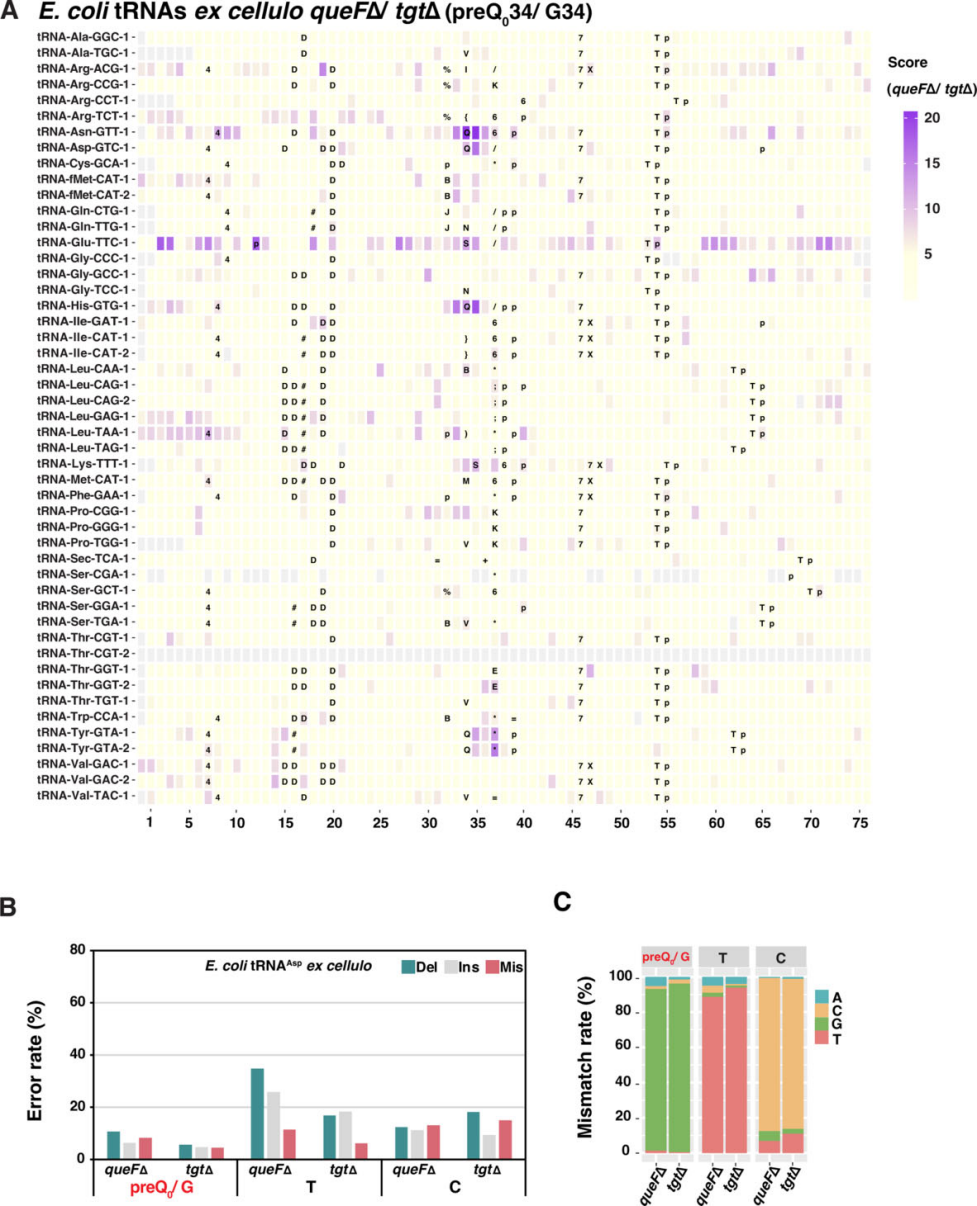
Detection of preQ_0_ in tRNAs from *E. coli*. (**A**) JACUSA2 scores of total tRNA from *E. coli queFΔ* versus *tgtΔ*. The position of each tRNA is aligned by anticodon, the first base of which is positioned 34. Representation as in Figure 4A. (**B**) Comparative analysis of error rates of tRNA^Asp^ from *E. coli queFΔ* versus *tgtΔ*. (**C)** Mismatch features of tRNA^Asp^ from *E. coli queFΔ* and *tgtΔ*.

In a third setup, we computed JACUSA2 scores for *queFΔ* compared *tgtΔ*, which is expected to reveal possible preQ_0_ modification of tRNAs. Intriguingly, we observed high scores for position 34 in tRNA^Asn^, tRNA^Asp^, and tRNA^His^, but not in tRNA^Tyr^ (Figure 6A). At face value, this suggests that the three former, but not the latter tRNA, are preQ_0_-modified in *queFΔ*. Notably, relatively high scores were present at the neighboring sites (position 35 to position 37) rather than at position 34 on tRNA^Tyr^. For position A37, this might be interpreted as a change in the level of ms^2^i^6^A37. However, this is difficult to reconcile with the notion that G34 is unmodified, since it then would be expected to be no different from A37 in tRNA^Tyr^ from *tgtΔ*.

Closer inspection of the error profiles of the four Q-tRNAs showed that error rates (especially deletions and mismatches) at position 35 were notably higher than at position 34, the presumed preQ_0_ modification site (Figure 6B and Supplementary Figure S15). As a consequence, the G to C misreading at position 34 in Q and preQ_1_-modified tRNAs was not seen in the *queFΔ* tRNAs (Figure 6C and Supplementary Figure S15). These results suggest that preQ_0_ is distinguishable from G in nanopore sequencing and base-calling, but the signatures are distinct from those of Q and preQ_1_. preQ_0_ modification appears to generate less stark error features, especially in tRNA^Tyr^, which may indicate that Q-tRNAs from the *queFΔ* strain are not fully preQ_0_-modified, and tRNA^Tyr^ may not be preQ_0_-modified.

**Figure 7.**
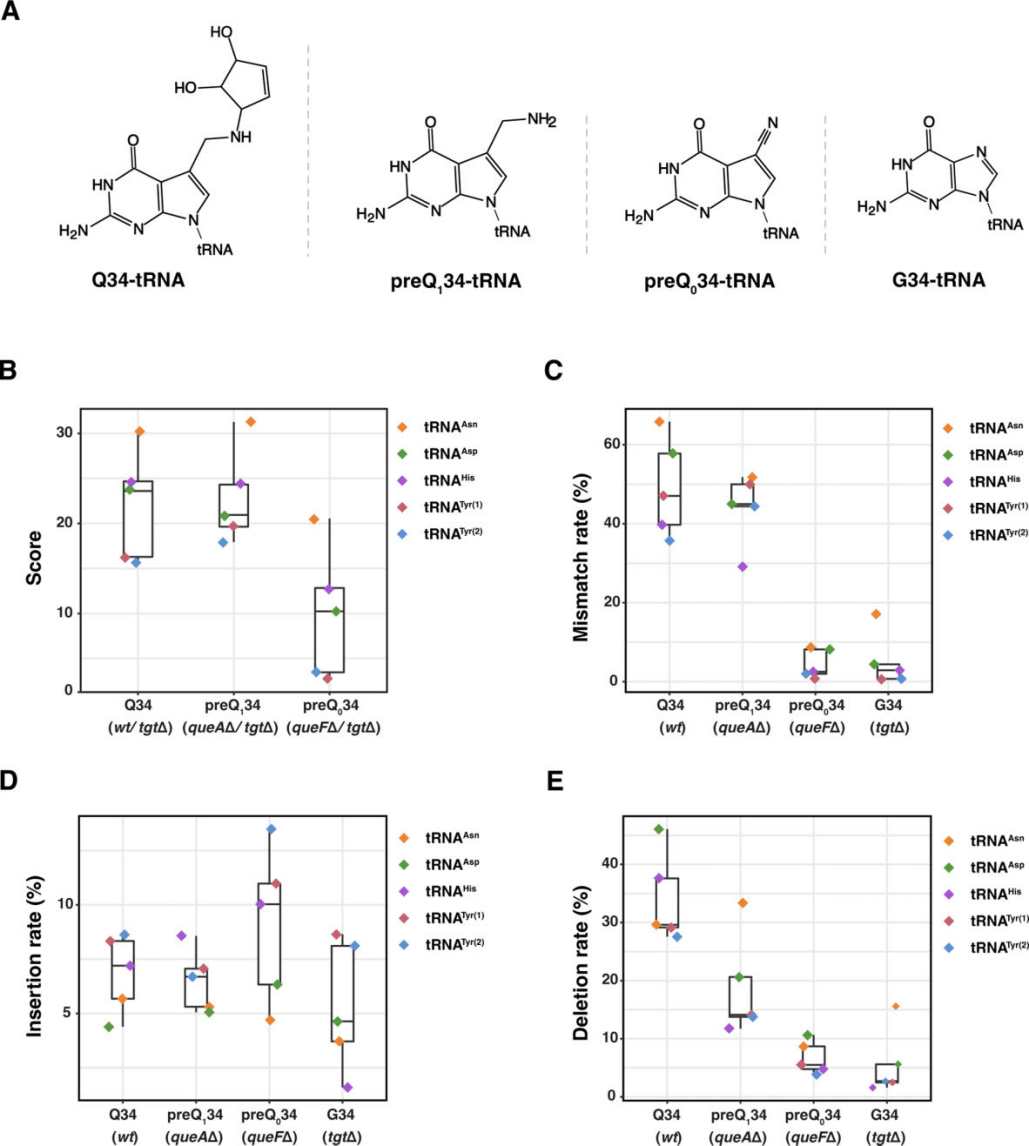
Detection of Q, preQ_1_, preQ_0_ in Q-tRNAs from *E. coli*. (**A**) Structures of Q, preQ_1_, preQ_0_ and G that are present at position 34 of Q-tRNAs. (**B**) JACUSA2 scores of Q34, preQ_1_34 and preQ_0_34 in Q-tRNAs. For detection of Q34, preQ_1_34 and preQ_0_34, JACUSA2 scores were computed for *wt* versus *tgtΔ*, *queAΔ* versus *tgtΔ*, and *queFΔ* versus *tgtΔ*, respectively. (**C**) Comparison of mismatch rates of Q34, preQ_1_34, preQ_0_34 and G34 in Q-tRNAs. (**D**) Comparison of insertion rates of Q34, preQ_1_34, preQ_0_34 and G34 in Q-tRNAs. (**E**) Comparison of deletion rates of Q34, preQ_1_34, preQ_0_34 and G34 in Q-tRNAs.

An overview of the JACUSA2 scores of Q/ preQ_1_/preQ_0_-modified positions in all four Q-tRNAs (Figure 7A) showed that the scores for Q and preQ_1_ at each position were higher than those for preQ_0_ (Figure 7B). While the scores, mismatch and insertion rates for Q and preQ_1_ were very similar, the deletion rates were lower for preQ_1_ (Figure 7C–E), indicating that the presence of the cyclopentene-diol moiety in Q increases deletion rates. For preQ_0_, there was a higher insertion rate (except for tRNA^Tyr^), whereas the mismatch and deletion rates were similar to those of G34 (*tgtΔ*). Thus, the 7-cyano-group in preQ_0_ increases insertion rates. Taken together, these results show that Q and Q precursors, including preQ_0_ and preQ_1_, are readily detectable and can be distinguished from each other by nanopore sequencing.

## Discussion

In this study, we have employed direct RNA sequencing to detect the 7-deaza-guanosine derivatives queuosine (Q), preQ_1_ and preQ_0_ in tRNAs by comparing read signatures from nanopore sequencing of unmodified to modified tRNAs using JACUSA2. We applied this to tRNAs obtained both by *in vitro* transcription as well as *ex cellulo* from *S. pombe* and *E. coli*. This approach allows the detection of these modifications at single-base resolution, and it overcomes limitations caused by other methods for Q detection, such as prior chemical treatment or an error-prone polymerase (46,48).

Specifically, using direct RNA sequencing, we found that Q modification resulted in elevated levels of sequencing errors (mismatches, deletions and insertions) at the Q34 position itself as well as at positions 5′ and 3′, thus generating a rather broad error profile. The error profiles at Q34 in *S. pombe* tRNA *ex cellulo* were similar to those of the corresponding *in vitro*-transcribed tRNAs, but varied with respect to the sequence context of Q34 in the different tRNAs. Of note, reliable detection of Q by direct sequencing requires the comparison between the modified and the non-modified state, for instance by comparing wild-type ‘without Q’ to ‘with Q’ samples, as a high rate of sequencing errors *per se* are insufficient to infer Q modification. Identifying partial Q modification levels seems generally possible, though using a framework to identify clusters of tRNAs with or without the modification would be the preferred method (60).

In principle, a goal could be to train a model to interpret the ionic currents (‘squiggles’) generated as individual bases pass through the nanopore. However, in this method, modified nucleotides generate distinct ionic current patterns within k-mers of sequence (generally 5 to 6 bases or more), rather than a unique pattern for a given nucleotide (61,62). Thus, training such a model would necessitate measuring Q modification in the context of every possible *k*-mer (*n* = 1024 for *k* = 5). However, this is not possible for Q, because it only exists in the four sequence contexts of the Q-tRNAs, such that generation of such a framework is neither possible nor necessary for Q using this approach.

The method used here is based on the ligation of adaptors to the tRNAs as described in Thomas *et al.* (51). In addition to those studies, we present here an improved method for the alignment of tRNA sequencing reads from modified tRNAs. First, we do not employ any heuristics such as the commonly used BWA software in the alignment process (63). Second, we use a statistically principled approach to assess the significance of tRNA alignments. In a direct comparison with the approach used in Lucas *et al.* (13) and using the exact same alignment scoring parameters, we observe fundamentally different alignments, as our approach yields higher scoring alignments. Moreover, we implement a data set specific dynamic alignment score cutoff at 95% specificity level by simulation. This constitutes another improvement over previous approaches (see Supplementary Materials and Methods). An improved overall alignment strategy is essential, since tRNAs are modified on average at 13 positions (4), the direct RNA sequencing yields reads with many mismatches compared to the reference sequence, which presents a challenge for heuristic alignment algorithms. We observed similar tRNA coverage of tRNAs in *E. coli* as the earlier work (51), thus showing good reproducibility, but there was only moderate correlation between coverage in *S. pombe* and the number of tRNA genes, which is generally taken as a measure for *in vivo* tRNA abundance. Thus, it appears that tRNA capture using the adaptors only poorly reflects tRNA levels in the cell, for reasons that are not apparent.

Next to Q, we were able to detect the Q precursors preQ_1_ (7-aminomethyl-deazaguanine) and preQ_0_ (7-cyano-7-deazaguanine) in tRNAs from *E. coli* by comparing direct RNA sequencing reads of *E. coli* strains with mutations in Q biosynthetic genes to those of a *tgtΔ* strain, where the respective tRNAs have G34. The precursors showed distinct error profiles, with Q showing the highest difference to G, followed by preQ_1_ and preQ_0_. Thus, the magnitude of sequencing errors and the breadth of the error peak correlates with the chemical structure of the modified bases, with the base with the largest difference to guanosine (Q) giving rise to the most distinct error profile. However, assignment of a specific modification is not possible as yet, unless the comparative information of the modified and unmodified state is available. Also, our method currently cannot distinguish between Q and its precursors within a single tRNA sample, since this would require its own computational framework (60). It should, however, in principle be possible to quantitate the modification level of a single 7-deazaguanine precursor (64). Of note, nanopore sequencing has been employed to determine preQ_0_ modification in the DNA of a phage (65).

In principle, it should be possible to identify any modified RNA base using direct RNA sequencing. Our direct RNA sequencing approach was able to pinpoint other tRNA modifications in the four Q-tRNAs by comparing *ex cellulo* tRNAs to the *in vitro*-transcribed tRNAs. High error scores were found at the known modification sites m^7^G, i^6^A, Gm, m^1^A, Ψ and D, though many other sites also showed high scores. It remains to be seen whether and how these other sites are modified, and some high scores might be the result of a broad error profile of a big chemical modification at a neighboring site. Conversely, some (smaller) modifications might be ‘hidden’ under the error profile of another site. As noted above, assigning a modification to a particular base will require comparisons between wild-type yeast or bacteria and strains with mutations or deletions of the respective genes encoding tRNA modification enzymes. The observation that some modifications (for instance Q) give rise to a broad error profile, whereas other modifications result in a sharper profile, may be useful for the training of networks for modification detection.

In our data of tRNAs *ex cellulo* from yeast and bacteria, it was also of interest to investigate potential crosstalk of Q (and Q precursors) with other tRNA modifications. The analysis suggested that no modifications other than Q34 change upon Q modification both in *S. pombe* and *E. coli*. However, it is notable that the known cross-talk between Q34 and m^5^C at C38 in tRNA^Asp^ in *S. pombe* (33) was not detected here (there is no m^5^C38 in tRNA^Asp^ in *E. coli*), possibly because C38 is too close to Q34 and is ‘hidden’ underneath its broad error profile. Another possibility is that m^5^C is too similar to the canonical C to result in a distinct error profile with the software currently available for interpretation of the ‘squiggles’, a notion that is echoed in earlier work (66). Thus, whether modification of sites other than m^5^C38 show cross-talk with Q awaits further analysis.

Overall, our results show that Q and Q precursors are readily detectable in tRNAs using direct RNA sequencing. The improved alignment approach allows for a more stringent and controlled assignment of sequence reads to individual tRNAs. Furthermore, the comparison of tRNA reads *ex cellulo* to *in vitro* transcripts indicates that this method, in principle, should be applicable to any tRNA modification and will be able to measure different modifications on the same tRNA species. Analysis of tRNAs from deletion mutants will allow the comprehensive mapping of tRNA modifications and may reveal new cross-modification cross-talks.

## Supplementary Material

gkad826_Supplemental_Files

## Data Availability

All details regarding alignment and JACUSA2 analysis can be found at https://doi.org/10.5281/zenodo.8325692. High-throughput screening data are available in the NCBI GEO database: record GSE236838.
