# Supplementary material for: Detection of queuosine and queuosine precursors in tRNAs by direct RNA sequencing
Source: Nucleic Acids Res. 2023 Oct 9;51(20):11197–212. doi: 10.1093/nar/gkad826 (PMC10639084; doi:10.1093/nar/gkad826)
Supplement: gkad826_Supplemental_Files [file gkad826_supplemental_files.zip › Supplementary_Materials.pdf]

SUPPLEMENTARY MATERIALS

Supplementary Materials and Methods

Comparison of alignment strategies

Previous work on tRNA sequencing on the ONT platform have used the BWA alignment algorithm (https://arxiv.org/abs/1303.3997) as a solution to produce local read alignments to a given reference database (1,2). BWA is a heuristic approach as it uses kmers for seeding alignments. The latest presented strategy computes alignments with the following BWA parameters:

```
bwa mem -t10 -W13 -k6 -xont2d -T20 (Lucas et al. 2023(1), STab 4)
```

Our alignment workflow employs an optimal local alignment strategy using the implementation as provided by the parasail software (3). The following parameter settings were used:

```
parasail_aligner -a sw_trace_striped_sse41_128_16 -M 1 -X 1 -e 1 -o
1 -c 20 -x -d -t 1 -O SAMH
```

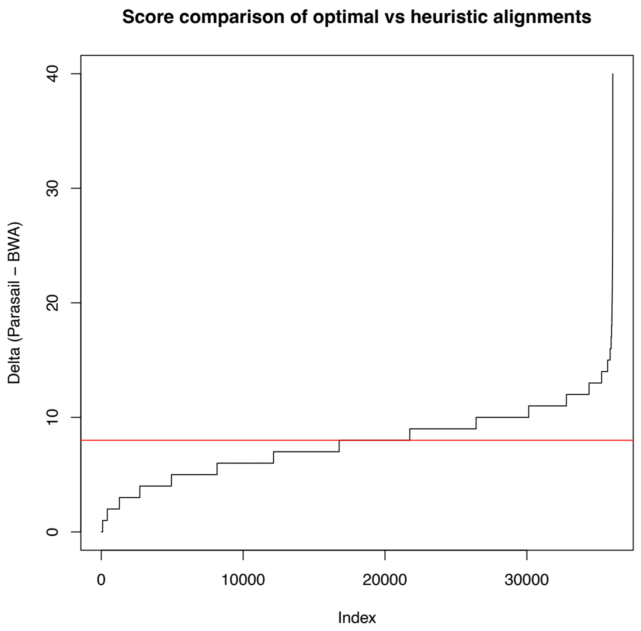

In essence, all alignments parameters e.g. mismatch penalties are identical for BWA and parasail. Using both strategies, we computed alignments for a wildtype S. pombe data set with 38,947 pass reads. Applying the same score cutoff of 20, we computed 38,127 parasail alignments and 36,055 bwa alignments. The read set for the latter overlaps by 100% with the parasail read set. Figure 1 (left) shows the distribution of score differences between parasail and BWA alignments. The median score difference (parasail – BWA) is +8-

An extreme example of diverging alignments across both strategies is shown below. Here, the same read (be281244-47bd-) gets assigned to two different tRNAs.

BWA alignment:

```
tRNA-Pro-TGG-2      1 CCTAAG-AGCAAGAAGAAGCCTGGGGGCTTCGTGGTGTAG-TGGTAGCAT
                      ||| .| |||..|||||||||| |||||.| | ||||.|| | ||  .||
be281244-47bd-     18 CCT-TGCAGCTGGAAGAAGCCT-GGGGCAT-G-GGTGCAGAT-GT--TAT

tRNA-Pro-TGG-2     49 ACTTCGTTTGG-GTCGAAGTGGTCAGGGGTTC-GATCCCCCTCGAAGC--
                       |.|| .|||| .|.||||    || .|...| |||||..|| |  ||
be281244-47bd-     61 -CGTC-CTTGGCATGGAAG----CA-TGCCCCAGATCCTGCT-G--GCTT

tRNA-Pro-TGG-2     95 --CCCCAGGCTTCTTC     108
                        |.||||||||||.||
be281244-47bd-    101 TGCACCAGGCTTCCTC     116

Length: 116, Identity: 73/116 (62.9%)., Similarity: 73/116 (62.9%), Gaps: 25/116 (21.6%),
Score: 30
```

parasail alignment:

```
tRNA-Ala-AGC-1       1 CCTAAG-AGCAAGAAGAAGCCTGGTGGGCATGTGGTGTAGATGGTTATCA
                         ||| .| |||..|||||||||||  ||||||| ||||.|||| |||||  
be281244-47bd-      18 CCT-TGCAGCTGGAAGAAGCCTG--GGGCATG-GGTGCAGAT-GTTAT--

tRNA-Ala-AGC-1      50 CGCTTCCTTAGCATGGAAG-AGGTCCCAGATTCGAGTTCTGGCTTGTCCA
                         ||  |||||.||||||||| |.|.|||||| ||  .|.||||||| |.||
be281244-47bd-      61 CG--TCCTTGGCATGGAAGCATGCCCCAGA-TC--CTGCTGGCTT-TGCA

tRNA-Ala-AGC-1      99 CCAGGCTTCTTC     110
                         |||||||||.||
be281244-47bd-     105 CCAGGCTTCCTC     116

Length: 112, Identity: 86/112 (76.8%), Similarity: 86/112 (76.8%), Gaps: 15/112 (13.4%),
Score: 60
```

In summary, optimal alignments may produce drastically different tRNA read mappings and are superior to heuristic alignments, which are the basis for modification calling.

Statistical assessment of alignment significance

Our strategy to assess the statistical significance is rooted in a simulation-based approach, which produces random alignments.

Briefly, we reverse input sequences. This obliterates the biological signal yet preserves nucleotide frequencies, which are the only relevant determinant in alignment scoring.

Then, we compute alignments in fwd orientation with all original reads and reverse orientation. We classify alignments in the fwd orientation as true and the ones with reverse read orientation as false.

Alignment precision is then defined by TP/(TP+FP) and alignment recall by TP/(TP+FN) according to some score threshold t. TP: true positive, FP: false positive and FN: false negative alignment relative to some score threshold t.

On the next page, we see a precision-recall curve that plots both performance measures into a single plot. The strategy of Lucas et al. uses a fixed score cutoff of 20, which yields an alignment set with less than 50% precision (red dot, blue arrow).

On the other hand, we are now in the position to dynamically set the score cutoff to e.g. a precision level of 95% for example (green arrow).

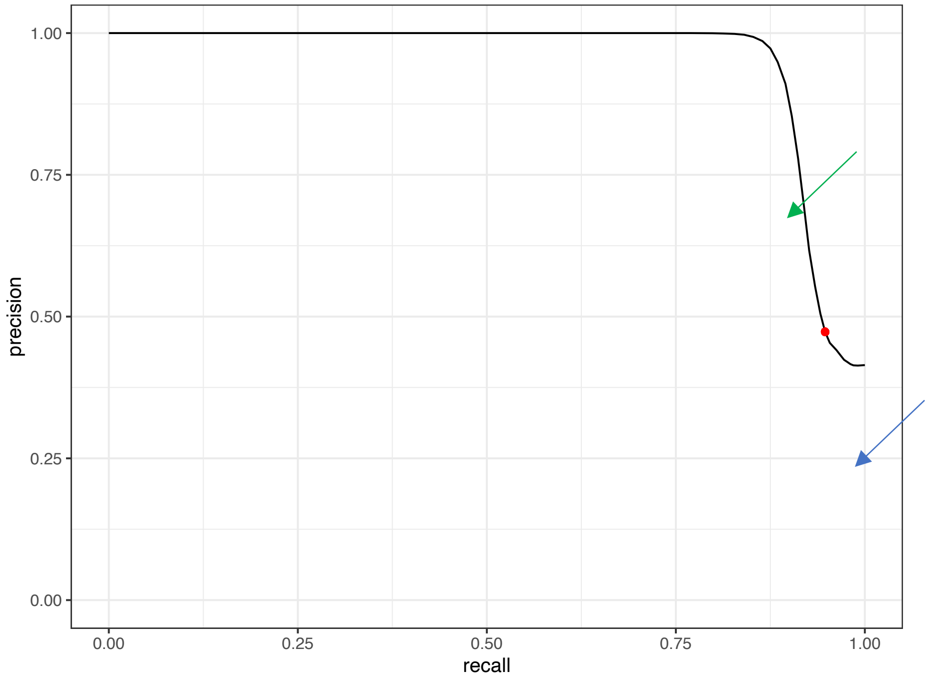

JACUSA2 analysis
All used R scripts have been deposited to: https://github.com/dieterich-lab/QutRNA.
The analysis has been conducted with R version 4.2.2.

Comparing different conditions with JACUSA2
JACUSA2 (version 2.0.4) was used to perform head-to-head comparisons between different conditions:

"jacusa2 call-2 -m 1 -q 1 -c 4 -p 2 -D -i -a D,Y -P1 FR-SECONDSTRAND -P2 FR-SECONDSTRAND \
-r <JACUSA2-OUTPUT> <CONDITION1.BAM> <CONDITION2.BAM>".

JACUSA2 output was processed with "JACUSA2_scores.R" to extract Mis+Del+Ins score, which is a sum of likelihood-ratio scores of JACUSA2 **Mis**(match), **Del**(etion), and **Ins**(ertion) scores (See (4) for details on the test-statistic):

"Rscript --vanilla JACUSA2_scores.R \
--fasta <FASTA> --mods <MODIFICATIONS> \
-o <JACUSA2_SCORE-OUTPUT> \
<JACUSA2-OUTPUT>".

Visualization of JACUSA2 score (Mis+Del+Ins)
Processed JACUSA2 output was visualized with "visualize_jacusa2_score.R":
"Rscript --vanilla visualize_jacusa2_score.R \
--left 24 --length 77 --prefix <PLOT-PREFIX> --score "Mis+Del+Ins" <JACUSA2_SCORE-OUTPUT>.

Supplementary References

1. Lucas, M.C., Pryszcz, L.P., Medina, R., Milenkovic, I., Camacho, N., Marchand, V., Motorin, Y., Ribas de Pouplana, L. and Novoa, E.M. (2023) Quantitative analysis of tRNA abundance and modifications by nanopore RNA sequencing. *Nat Biotechnol*.
2. Leger, A., Amaral, P.P., Pandolfini, L., Capitanchik, C., Capraro, F., Miano, V., Migliori, V., Toolan-Kerr, P., Sideri, T., Enright, A.J. *et al.* (2021) RNA modifications detection by comparative Nanopore direct RNA sequencing. *Nat Commun*, **12**, 7198.
3. Daily, J. (2016) Parasail: SIMD C library for global, semi-global, and local pairwise sequence alignments. *BMC Bioinformatics*, **17**, 81.
4. Piechotta, M., Naarmann-de Vries, I.S., Wang, Q., Altmuller, J. and Dieterich, C. (2022) RNA modification mapping with JACUSA2. *Genome Biol*, **23**, 115.

Supplementary Table S1: Plasmids used in this study.

Designation	Description	Source
pAE1688	pJET1-T7-tRNA^{Asp} (*S.pombe*)	Lab stock
pAE2240	pJET1-T7-tRNA^{His} (*S.pombe*)	Lab stock
pAE3371	pJET1-T7-tRNA^{Tyr} (*S.pombe*)	Lab stock
pAE3376	pJET1-T7-tRNA^{Asn} (*S.pombe*)	Lab stock
pAE3633	pJET1-T7-tRNA^{Asp} with 'CCA' (*S.pombe*)	This study
pAE3634	pJET1-T7-tRNA^{His} with 'CCA' (*S.pombe*)	This study
pAE3635	pJET1-T7-tRNA^{Tyr} with 'CCA' (*S.pombe*)	This study
pAE3636	pJET1-T7-tRNA^{Asn} with 'CCA' (*S.pombe*)	This study

Supplementary Table S2: Oligonucleotides used in this study.

Designation	Sequence *	Purpose
tRNAAsp-F	5'-GATGGCTCGAGTTTTTCAGCAAGATTT GTAATACGACTCACTATAGTCTCCT-3'	Plasmid construction
tRNAAsp-R	5'- GAACATCGATTTTCATGCATGGCGTCTCCC-3'	Plasmid construction
tRNAAsn-F	5'-GATGGCTCGAGTTTTTCAGCAAGATTT GTAATA CGACTCACTATAGGTCGG-3'	Plasmid construction
tRNAAsn-R	5'-GAACATCGATTTTCATGCATGGCGGTCAG-3'	Plasmid construction
tRNATyr-F	5'-GATGGCTCGAGTTTTTCAGCAAGATTT GTA ATACGACTCACTATAGCTCCTG-3'	Plasmid construction
tRNATyr-R	5'-GAACATCGATTTTCATGCATGGTGCTCCTG-3'	Plasmid construction
tRNAHis-F	5'-GATGGCTCGAGTTTTTCAGCAAGATTT GTA ATACGACTCACTATAGCTCAC-3'	Plasmid construction
tRNAHis-R	5'-GAACATCGATTTTCATGCATGGTGCCCAC-3'	Plasmid construction
tRNAAsp_probe	5'-biotin-GGGCTGCAAGCGTGACAGG-3'	Northern Blotting

E.coli_tgt_knockout.fwd	5'-CGCTGGTTTAAAACGTTGGACTGTTTTTCT GACGTAGTGGAGAAAAAATGCGATTGTGTAG GCTGGAGCTGCTTC-3'	Strain construction
E.coli_tgt_knockout.rev	5'-AGCTCATTAAATTTCCCTCATTATTAATATT AATCAACGTTCAAAGGTGGATGGGAATTAGC CATGGTCCATA -3'	Strain construction
3′-splint adapter	5'-Phosphate-ggcuucTTCTTGCTCTTAGGT AGTAGGTTC-3'	library preparation
5′-splint adapter-c	5'-Phosphate-CCTAAGagcaagaagaagccuggc-3'	library preparation
5′-splint adapter-a	5'-Phosphate-CCTAAGagcaagaagaagccugga-3'	library preparation
5′-splint adapter-g	5'-Phosphate-CCTAAGagcaagaagaagccuggg-3'	library preparation
5′-splint adapter-u	5'-Phosphate-CCTAAGagcaagaagaagccuggu-3'	library preparation

* lower-case letters denote ribonucleotides

Supplementary Table S3: *E. coli* strains used in this study.

Designation	Genotype	Source
AEB31	F- Δ(araD-araB)567, ΔlacZ4787 (::rrn B-3), λ–, rph-1, Δ(rhaD-rhaB)568, hsdR5154	CGSC (BW25113)
AEB26	F-, Δ(araD-araB)567, ΔlacZ4787(::rrnB-3), ΔqueA769::kan, λ-, rph-1, Δ(rhaD-rhaB)568, hsdR514	CGSC (JW0395-2)
AEB28	F-, Δ(araD-araB)567, ΔlacZ4787(::rrnB-3), λ-, ΔqueF721::kan, rph-1, Δ(rhaD-rhaB)568, hsdR514	CGSC (JW2765-1)
AEB36	F-, Δ(araD-araB)567, ΔlacZ4787(::rrnB-3), Δtgt::kan, λ-, rph-1, Δ(rhaD-rhaB)568, hsdR514	This study

Supplementary Figures

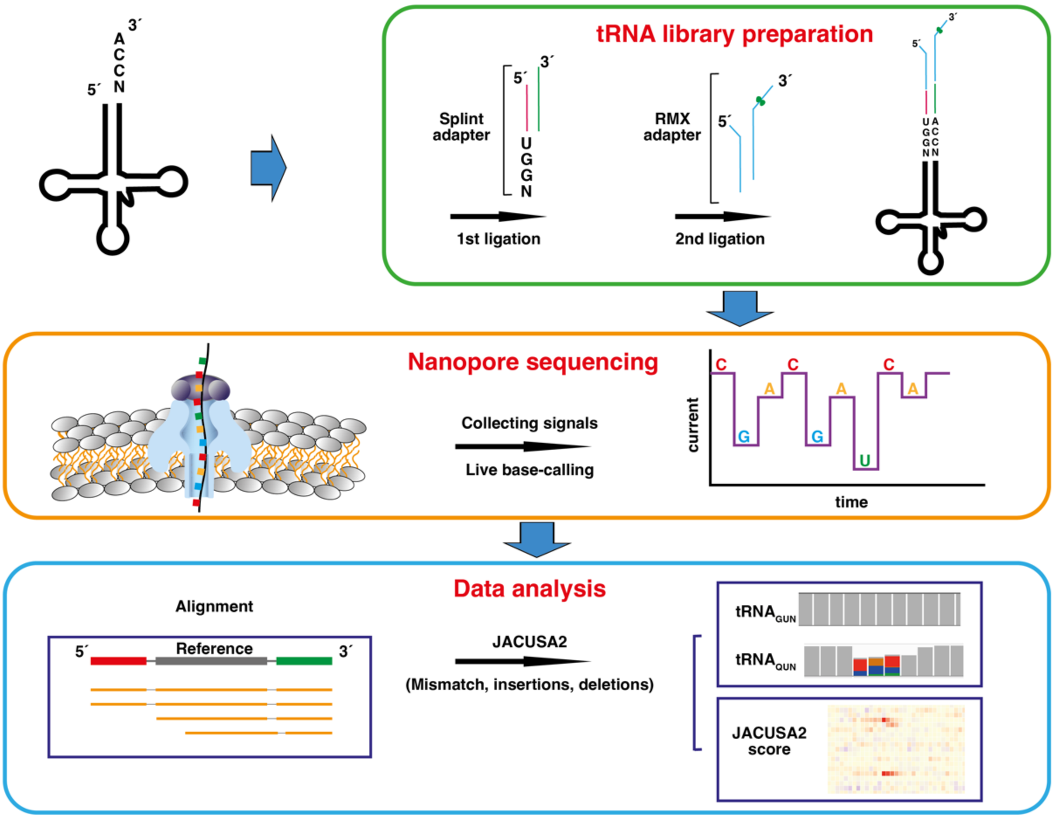

Supplementary Figure S1. Strategy for the detection of queuosine (Q) and Q precursors in tRNAs by direct RNA sequencing. tRNA molecules are ligated to double-stranded splint adapters, and the purified ligation product is ligated to the RMX adapters. Then, adapter-ligated tRNA is subjected to direct RNA sequencing with live base-calling on, and the sequencing reads are aligned to the reference composed of splint adapters and tRNA sequence. JACUSA2 is employed to detect mismatch, insertions and deletions, and assigns score to each tRNA position based on the comparison between tRNAs with and without Q/ Q precursor modification. High JACUSA2 scores are indicative of a modification at the respective positions.

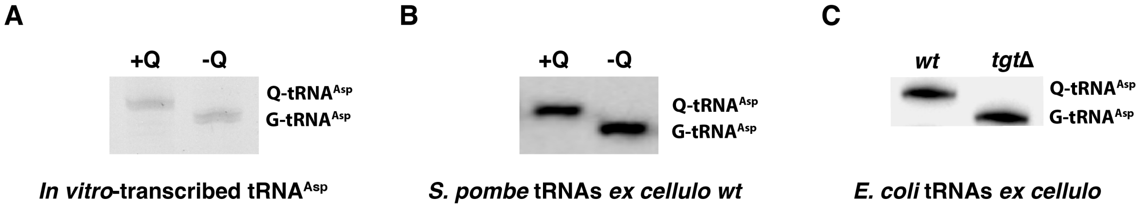

Supplementary Figure S2. Analysis of Q modification levels in *in vitro*-transcribed tRNA and *ex cellulo* tRNA. (**A**) Measurement of Q levels in *in vitro*-transcribed $tRNA^{Asp}$ with and without Q modification (introduced with hTGT) using polyacrylamide gels covalently linked with N-acryloyl-3-aminophenylboronic acid (APB). (**B**) Measurement of Q levels in *S. pombe* $tRNA^{Asp}$ *ex cellulo* by Northern blotting with APB-gels. Small RNA samples from wild-type *S. pombe* cells cultivated in the presence (+Q) or absence (-Q) of queuine are shown. (**C**) Measurement of Q levels in *E. coli* $tRNA^{Asp}$ *ex cellulo* by Northern blotting with APB-gels. Small RNA samples from *E. coli wt* and *tgt*Δ are shown.

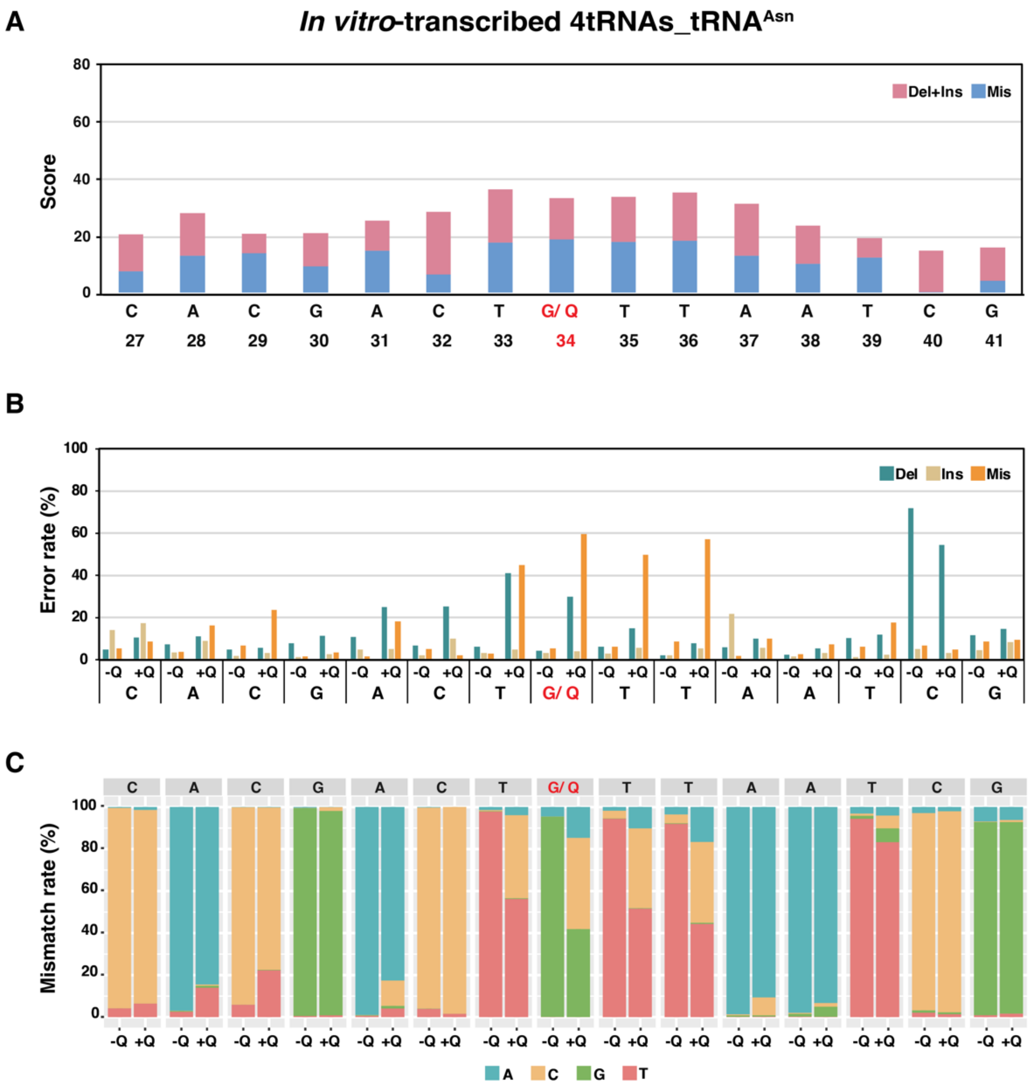

Supplementary Figure S3. Detection of Q modification on *in vitro*-transcribed $tRNA^{Asn}$ by nanopore direct RNA sequencing. (**A**) JACUSA2 scores of Q34 and the surrounding nucleotides in $tRNA^{Asn}$. The scores are derived from JACUSA2 call-2 analysis of *in vitro*-transcribed $tRNA^{Asn}$ with (+Q) and without (-Q) Q-modification, considering deletions and insertions (pink) as well as mismatches (blue). (**B**) Comparison of error rates (deletions, insertions and mismatch rates) of *in vitro*-transcribed $tRNA^{Asn}$ +/ -Q on the same sites as in **A**. The error rates were calculated by JACUSA2. (**C**) Comparison of mismatch signature from *in vitro*-transcribed $tRNA^{Asn}$ +/ -Q. At each position, the frequencies of individual bases as given by JACUSA2 are shown.

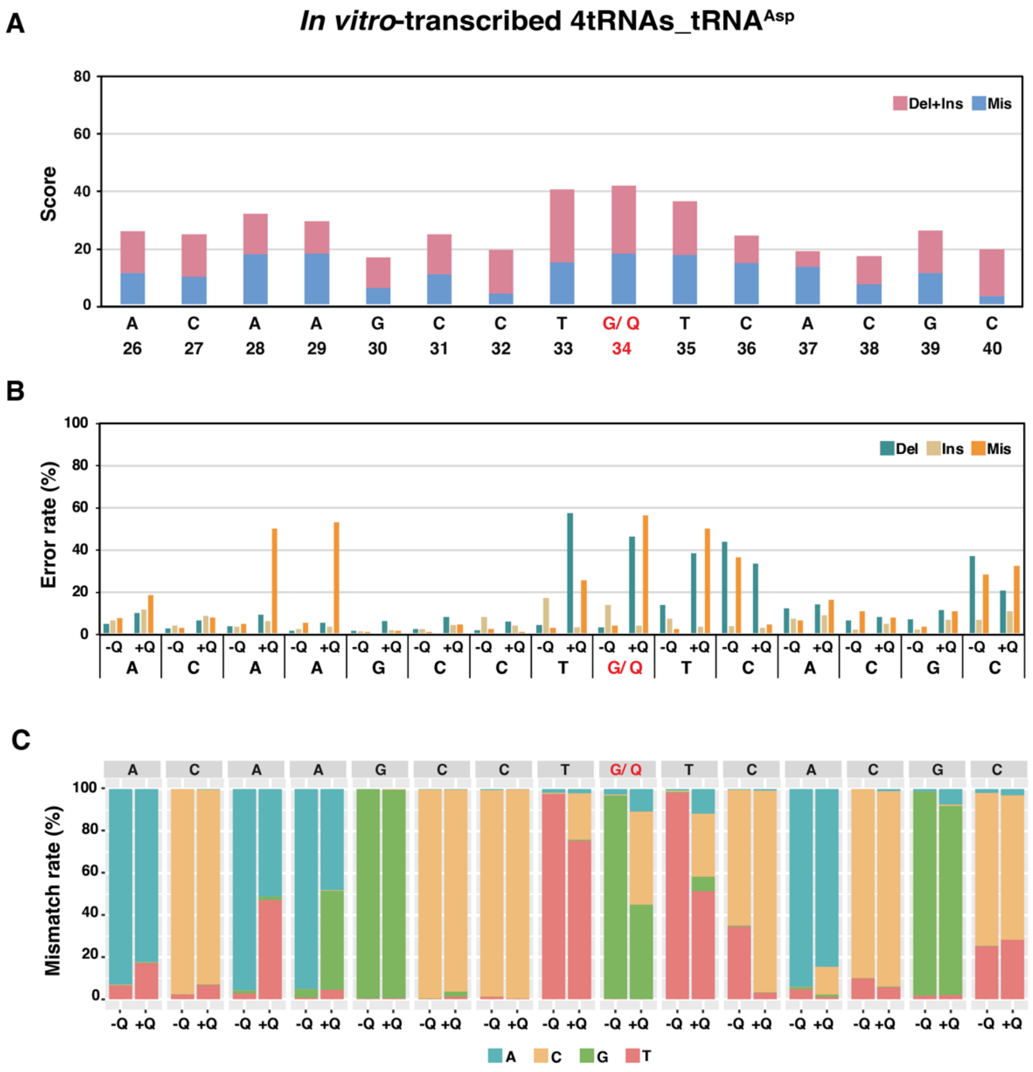

Supplementary Figure S4. Detection of Q modification on *in vitro*-transcribed tRNA^{Asp} by nanopore direct RNA sequencing. (**A**) JACUSA2 scores of Q34 and the surrounding nucleotides in tRNA^{Asp}. The scores are derived from JACUSA2 call-2 analysis of *in vitro*-transcribed tRNA^{Asp} with (+Q) or without (-Q) Q-modification. (**B**) Comparison of error rates of *in vitro*-transcribed tRNA^{Asp} +/ -Q on the same sites as in **A**. (**C**) Comparison of mismatch signature from *in vitro*-transcribed tRNA^{Asp} +/ -Q.

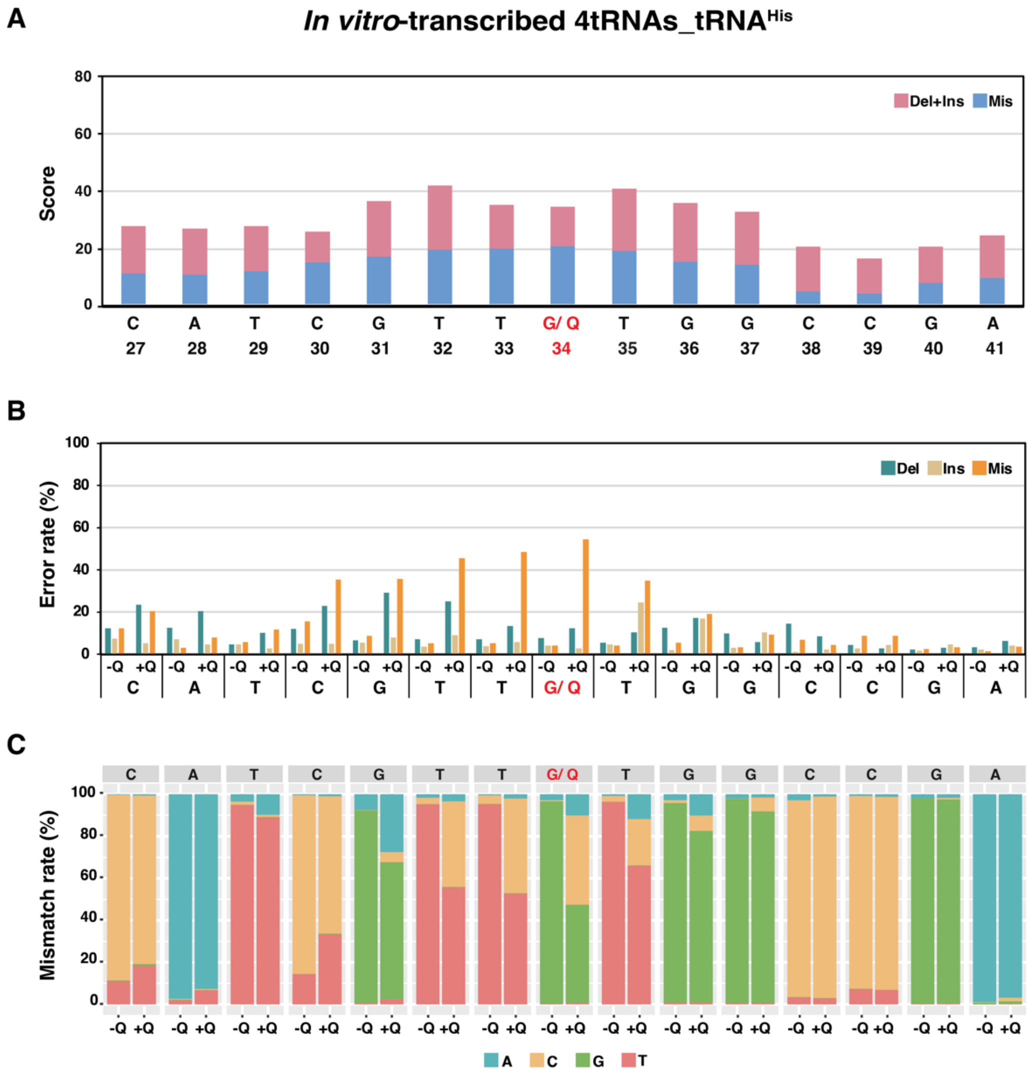

Supplementary Figure S5. Detection of Q modification on *in vitro*-transcribed tRNA^{His} by nanopore direct RNA sequencing. (**A**) JACUSA2 scores of Q34 and the surrounding nucleotides in tRNA^{His}. The scores are derived from JACUSA2 call-2 analysis of *in vitro*-transcribed tRNA^{His} with (+Q) or without (-Q) Q-modification. (**B**) Comparison of error rates of *in vitro*-transcribed tRNA^{His} +/ -Q on the same sites as in **A**. (**C**) Comparison of mismatch signature from *in vitro*-transcribed tRNA^{His} +/ -Q.

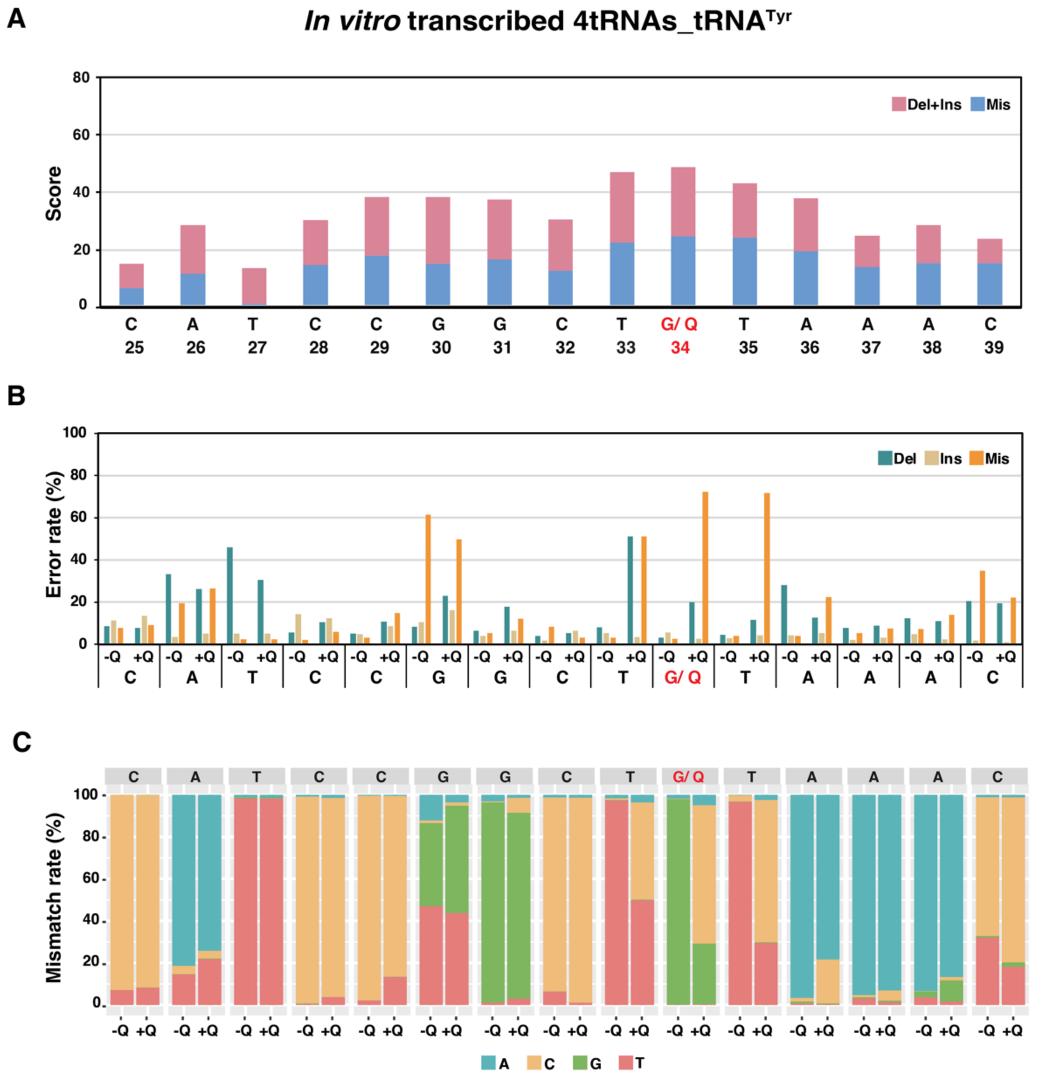

Supplementary Figure S6. Detection of Q modification on *in vitro*-transcribed tRNA^{Tyr} by nanopore direct RNA sequencing. (**A**) JACUSA2 scores of Q34 and the surrounding nucleotides in tRNA^{Tyr}. The scores are derived from JACUSA2 call-2 analysis of *in vitro*-transcribed tRNA^{Tyr} with (+Q) or without (-Q) Q-modification. (**B**) Comparison of error rates of *in vitro*-transcribed tRNA^{Tyr} +/ -Q on the same sites as in **A**. (**C**) Comparison of mismatch signature from *in vitro*-transcribed tRNA^{Tyr} +/ -Q.

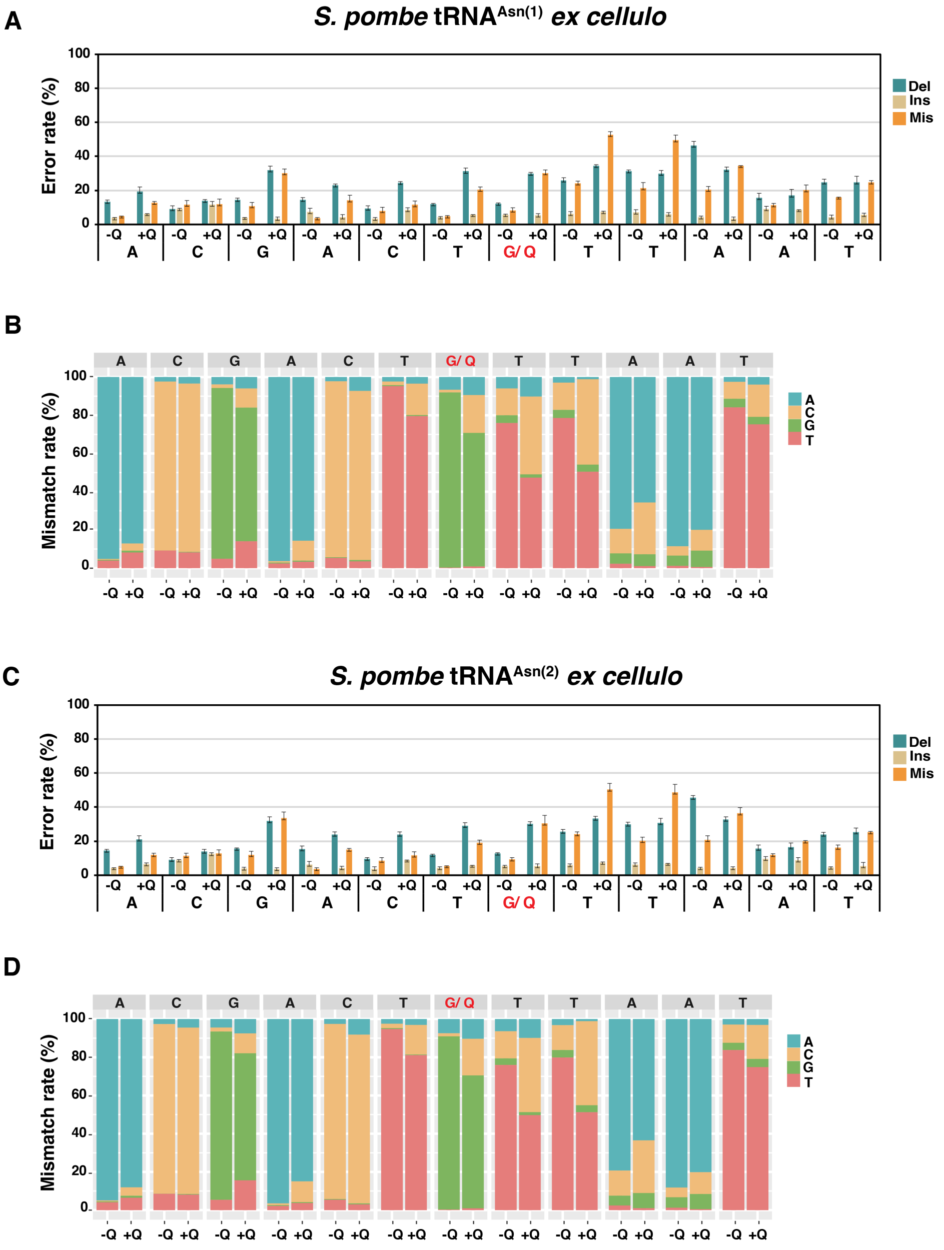

Supplementary Figure S7. Detection of Q modification on *S. pombe* $tRNA^{Asn}$ *ex cellulo* by nanopore direct RNA sequencing. (**A**) Comparison of error rates (deletions, insertions and mismatch rates) of $tRNA^{Asn(1)}$ from *S. pombe wt* cells cultivated in the absence (*wt*-Q) and presence (*wt*+Q) of queuine. The values are the average of three biological replicates per condition, and the error bars represent standard deviations. (**B**) Misincorporation signature of $tRNA^{Asn(1)}$ from *S. pombe wt* -/ +Q. At each position, the frequencies of individual bases as given by JACUSA2 are shown. (**C**) Comparison of error rates of $tRNA^{Asn(2)}$ from *S. pombe wt* -/ +Q. The values are the average of three biological replicates per condition, and the error bars represent standard deviations. (**D**) Misincorporation signature of $tRNA^{Asn(2)}$ from *S. pombe wt* -/ +Q.

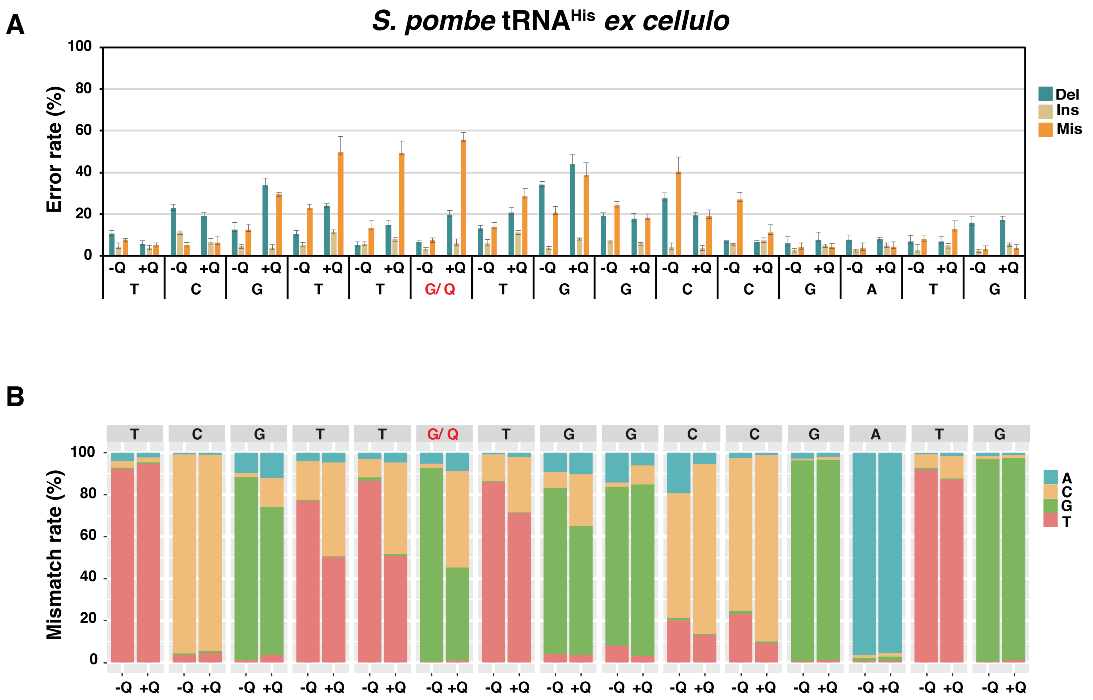

Supplementary Figure S8. Detection of Q modification on *S. pombe* $tRNA^{His}$ *ex cellulo* by nanopore direct RNA sequencing. (**A**) Comparison of error rates of $tRNA^{His}$ from *S. pombe wt* cells cultivated in the absence (*wt*-Q) and presence (*wt*+Q) of queuine. The values are the average of three biological replicates per condition, and the error bars represent standard deviations. (**B**) Misincorporation signature of $tRNA^{His}$ from *S. pombe wt* -/ +Q. At each position, the frequencies of individual bases as given by JACUSA2 are shown.

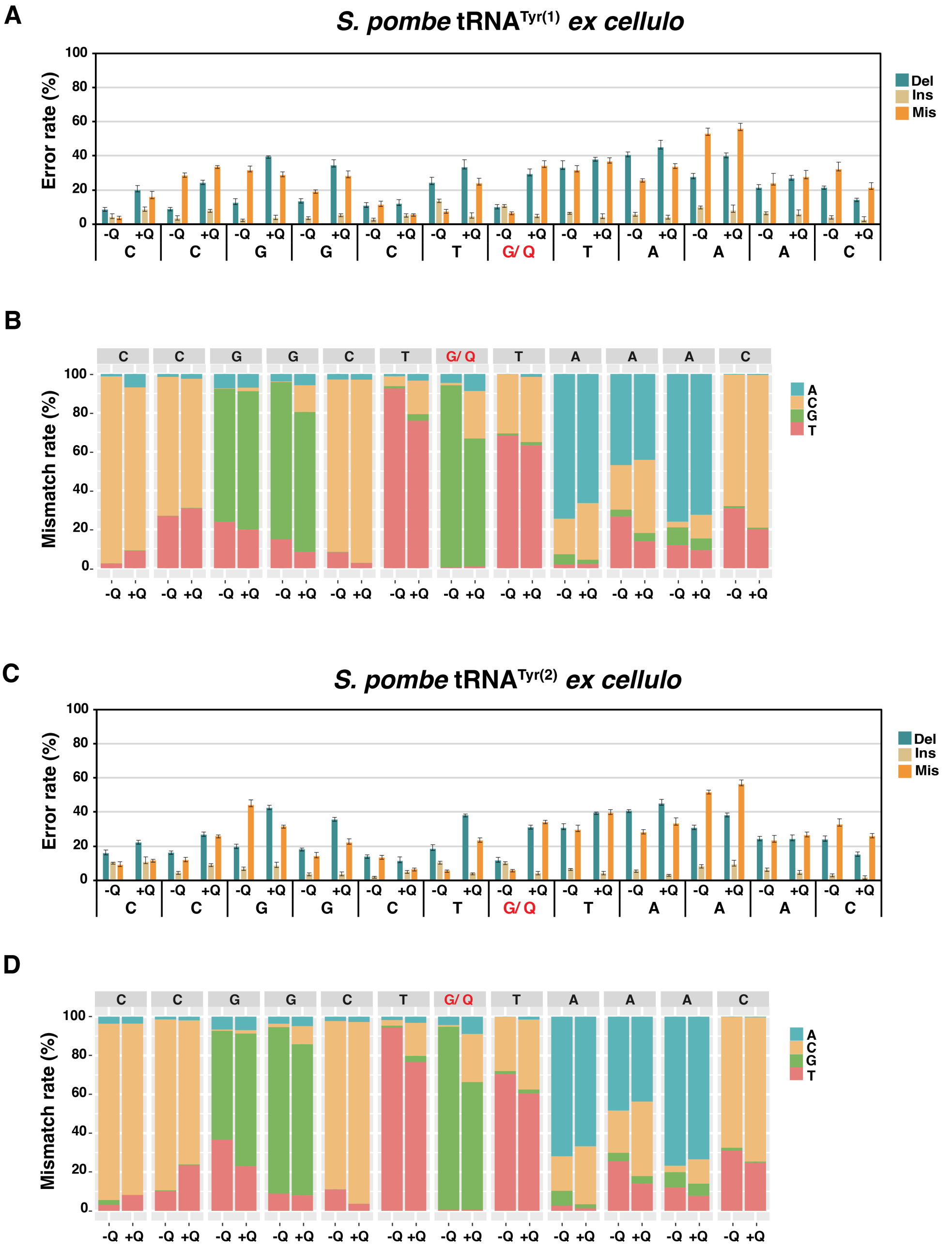

Supplementary Figure S9. Detection of Q modification on *S. pombe* tRNA^{Tyr} *ex cellulo* by nanopore direct RNA sequencing. (**A**) Comparison of error rates of tRNA$^{Tyr(1)}$ from *S. pombe wt* cells cultivated in the absence (*wt*-Q) and presence (*wt*+Q) of queuine. The values are the average of three biological replicates per condition, and the error bars represent standard deviations. (**B**) Misincorporation signature of tRNA$^{Tyr(1)}$ from *S. pombe wt* -/ +Q. At each position, the frequencies of individual bases as given by JACUSA2 are shown. (**C**) Comparison of error rates of tRNA$^{Tyr(2)}$ from *S. pombe wt* -/ +Q. The values are the average of three biological replicates per condition, and the error bars represent standard deviations. (**D**) Misincorporation signature of tRNA$^{Tyr(2)}$ from *S. pombe wt* -/ +Q.

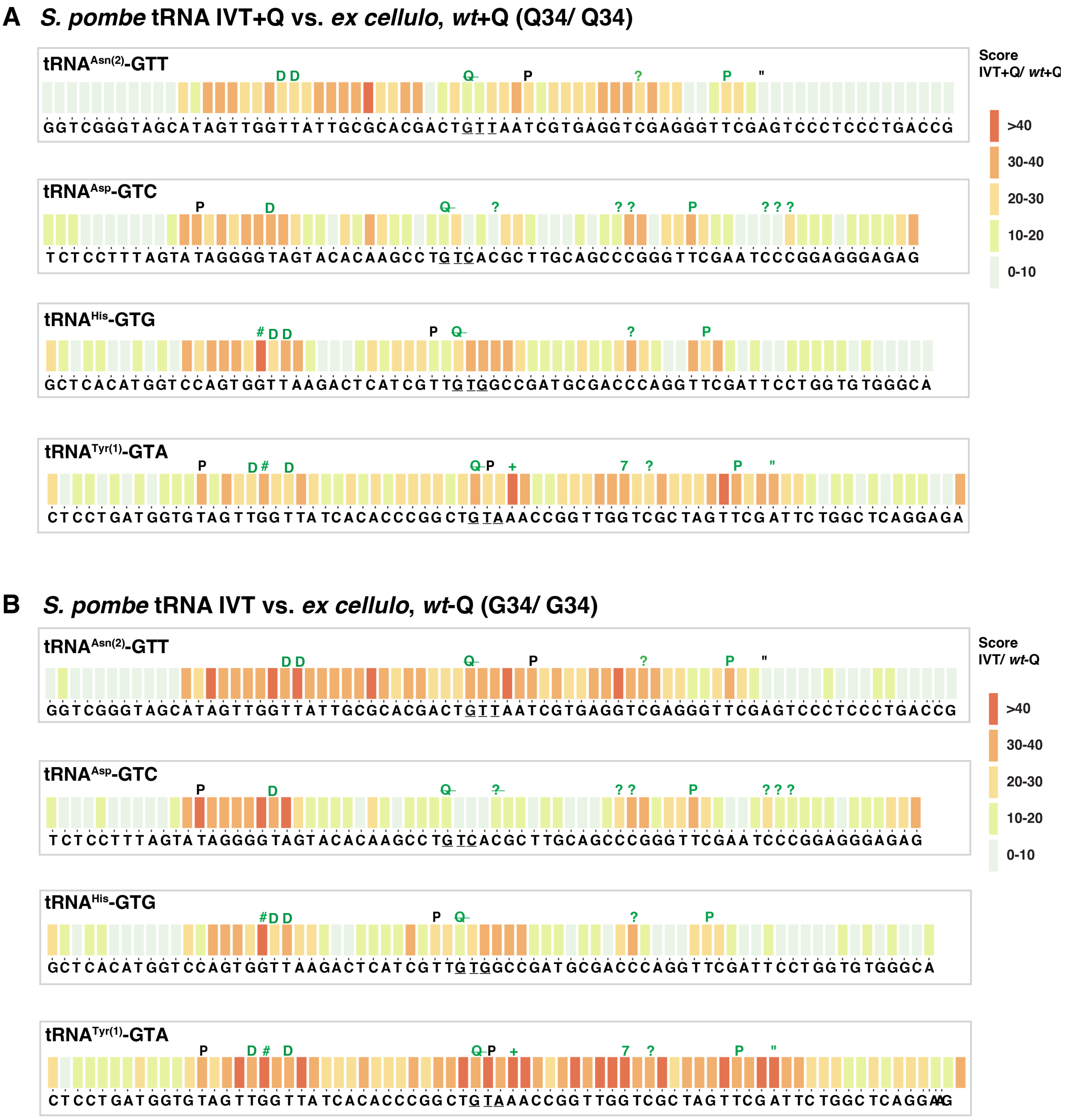

Supplementary Figure S10. Detection of tRNA modifications in *S. pombe* by comparison of nanopore sequencing errors in tRNAs *ex cellulo* to *in vitro*-transcribed tRNAs. (**A**) JACUSA2 score of comparison between *in vitro*-transcribed tRNAs with Q modification (IVT+Q) and the corresponding tRNAs from *S. pombe wt* cells cultivated in the presence of queuine (*wt*+Q). Known (green) and predicted (black) modification sites are indicated above the columns. (**B**) JACUSA2 score of comparison between *in vitro*-transcribed tRNAs (IVT) and the corresponding tRNAs from *S. pombe wt* cells cultivated in the absence of queuine (*wt*-Q).

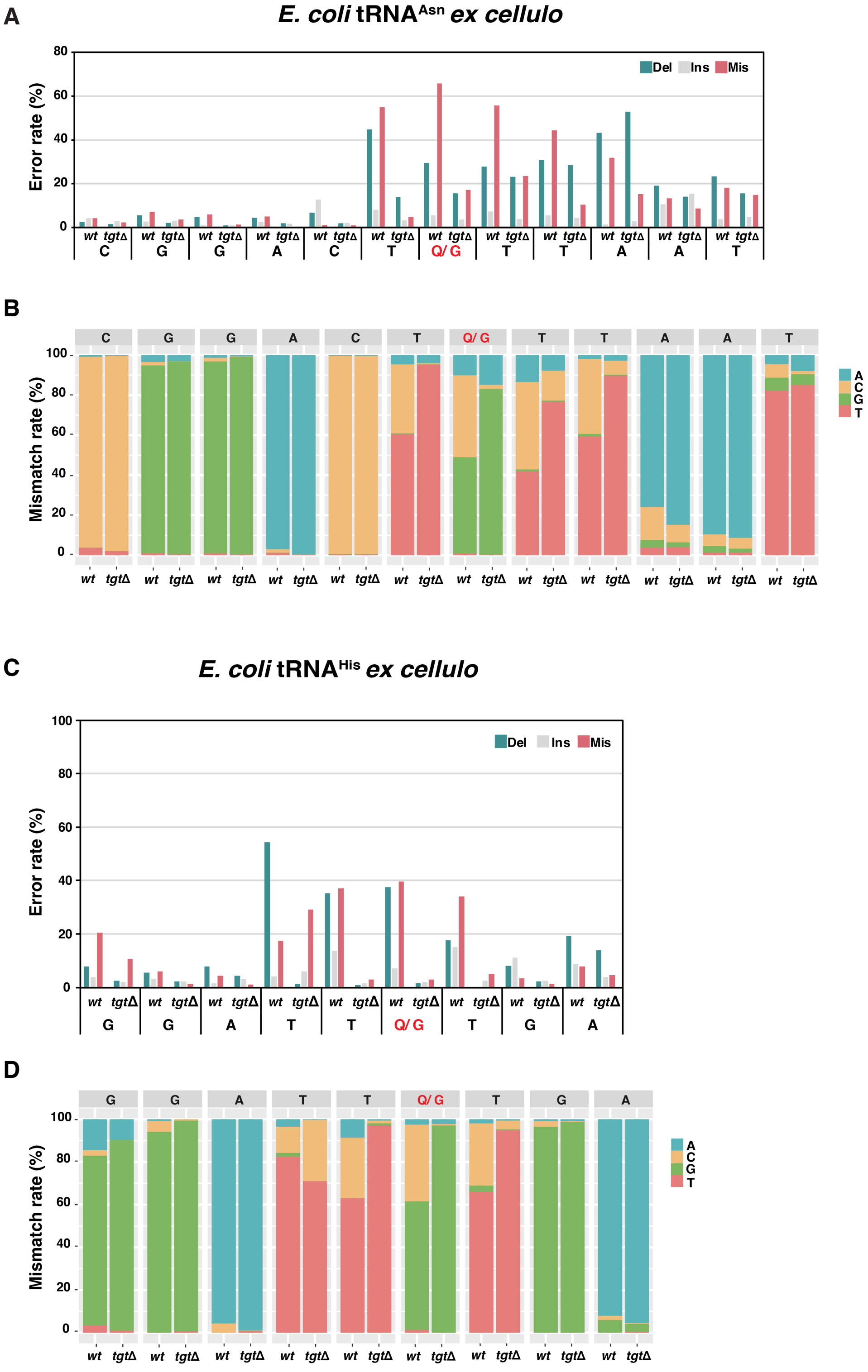

Supplementary Figure S11. Detection of Q modification in tRNA^{Asn} and tRNA^{His} from *E. coli* by direct RNA sequencing. (**A**) Comparative analysis of error rates of tRNA^{Asn} from *E. coli wt* versus *tgt*Δ. (**B**) Mismatch features of tRNA^{Asn} from *E. coli wt* and *tgt*Δ. (**C**) Comparative analysis of error rates of tRNA^{His} from *E. coli wt* versus *tgt*Δ. (**D**) Mismatch features of tRNA^{His} from *E. coli wt* and *tgt*Δ.

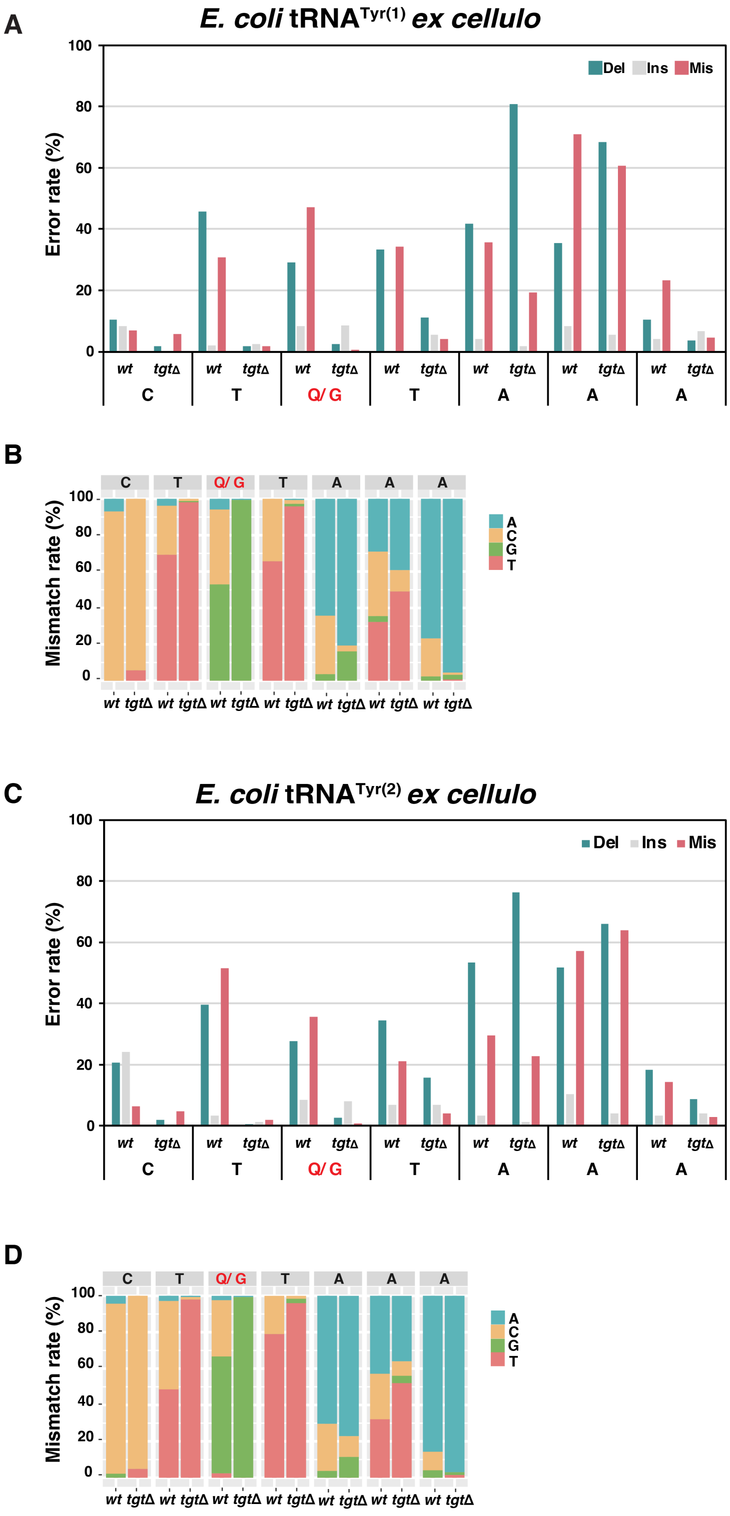

Supplementary Figure S12. Detection of Q modification in tRNA^{Tyr} from *E. coli* by direct RNA sequencing. (**A**) Comparative analysis of error rates of tRNA$^{Tyr(1)}$ from *E. coli wt* versus *tgt*Δ. (**B**) Mismatch features of tRNA$^{Tyr(1)}$ from *E. coli wt* and *tgt*Δ. (**C**) Comparative analysis of error rates of tRNA$^{Tyr(2)}$ from *E. coli wt* versus *tgt*Δ. (**D**) Mismatch features of tRNA$^{Tyr(2)}$ from *E. coli wt* and *tgt*Δ.

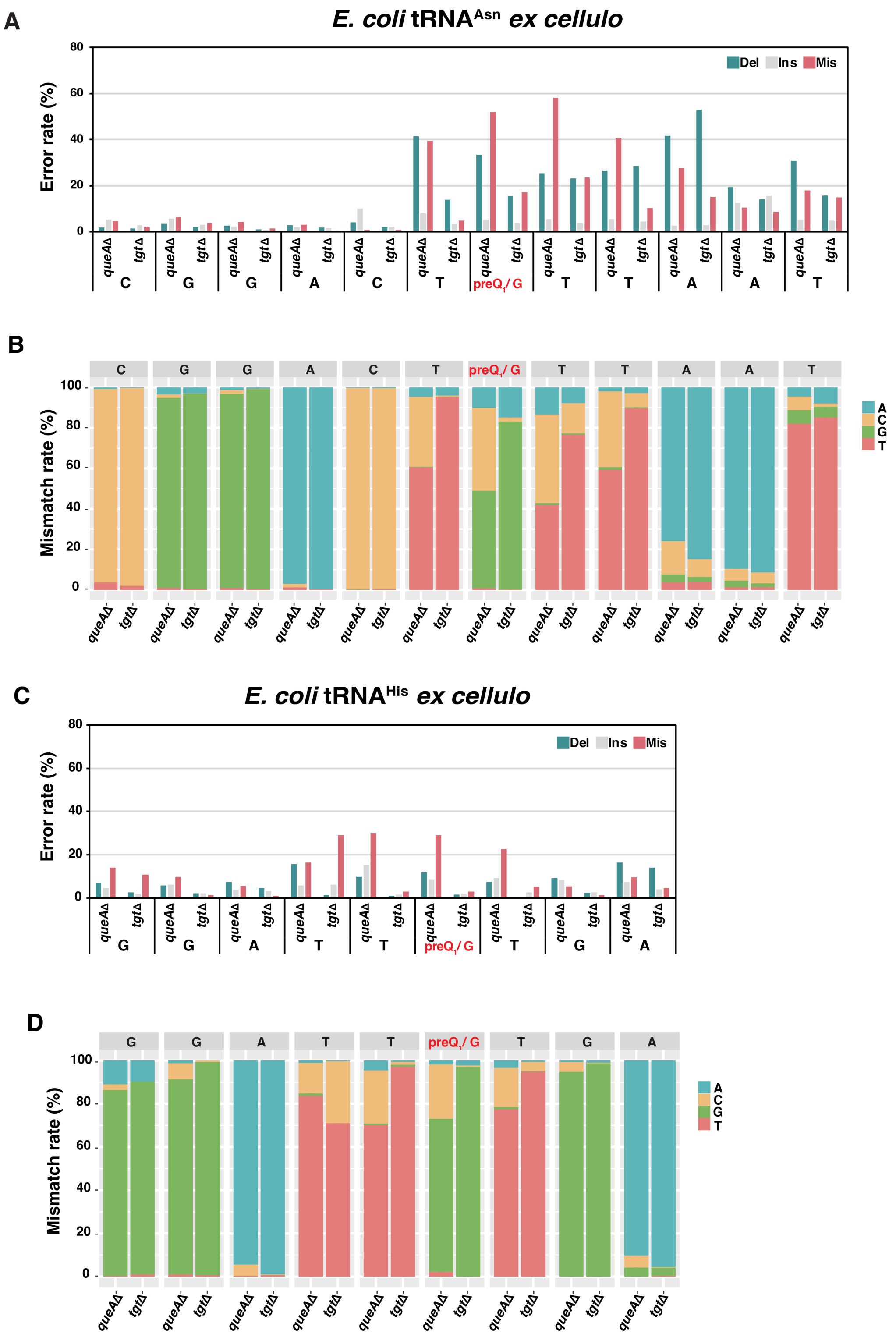

Supplementary Figure S13. Detection of $preQ_1$ in $tRNA^{Asn}$ and $tRNA^{His}$ from *E. coli* by direct RNA sequencing. (**A**) Comparative analysis of error rates of $tRNA^{Asn}$ from *E. coli queA*Δ versus *tgt*Δ. (**B**) Mismatch features of $tRNA^{Asn}$ from *E. coli queA*Δ and *tgt*Δ. (**C**) Comparative analysis of error rates of $tRNA^{His}$ from *E. coli queA*Δ versus *tgt*Δ. (**D**) Mismatch features of $tRNA^{His}$ from *E. coli queA*Δ and *tgt*Δ.

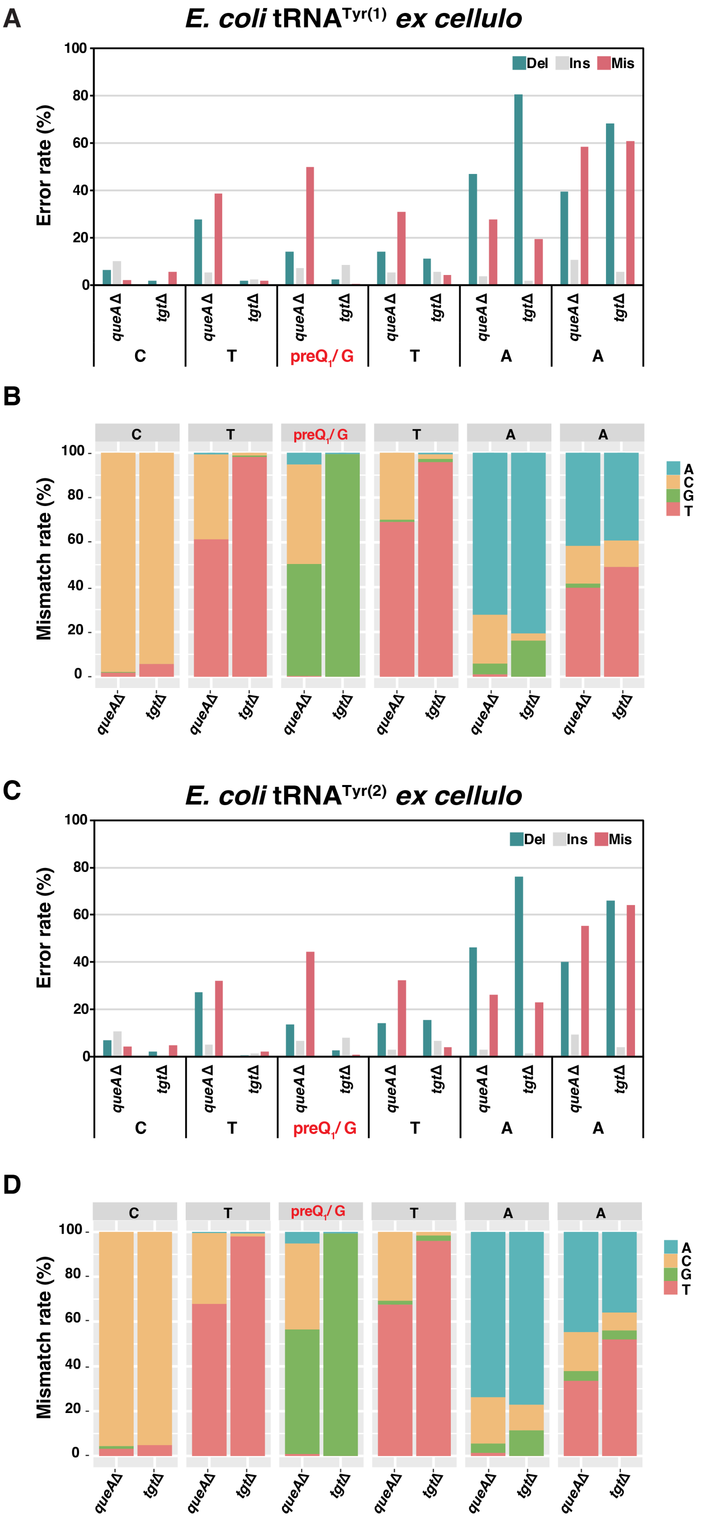

Supplementary Figure S14. Detection of $preQ_1$ in $tRNA^{Tyr}$ from *E. coli* by direct RNA sequencing. (**A**) Comparative analysis of error rates of $tRNA^{Tyr(1)}$ from *E. coli queA*Δ versus *tgt*Δ. (**B**) Mismatch features of $tRNA^{Tyr(1)}$ from *E. coli queA*Δ and *tgt*Δ. (**C**) Comparative analysis of error rates of $tRNA^{Tyr(2)}$ from *E. coli queA*Δ versus *tgt*Δ. (**D**) Mismatch features of $tRNA^{Tyr(2)}$ from *E. coli queA*Δ and *tgt*Δ.

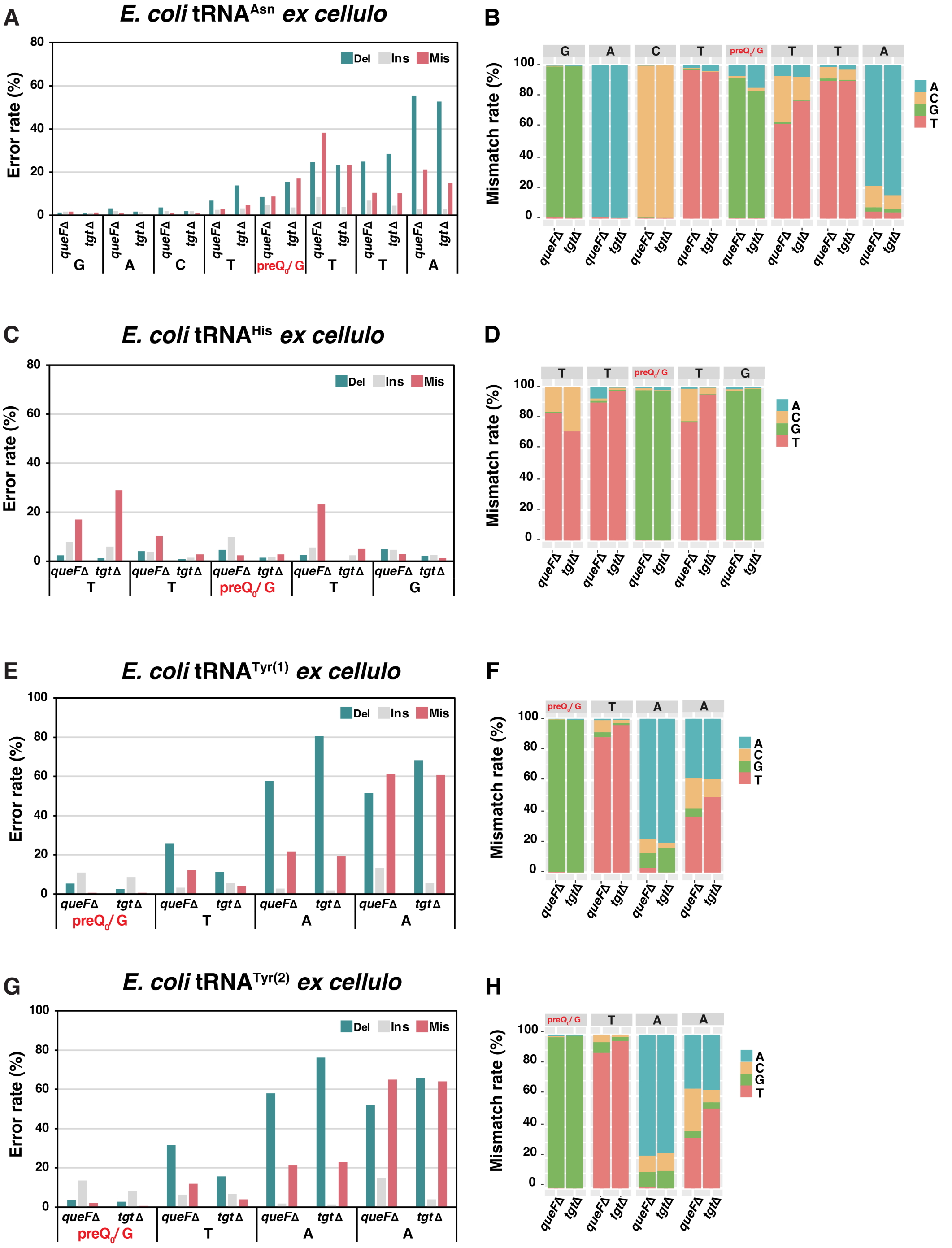

Supplementary Figure S15. Detection of preQ$_0$ in tRNAs from *E. coli* by direct RNA sequencing. (**A**) Comparative analysis of error rates of tRNA^{Asn} from *E. coli queF*Δ versus *tgt*Δ. (**B**) Mismatch features of tRNA^{Asn} from *E. coli queF*Δ and *tgt*Δ. (**C**) Comparative analysis of error rates of tRNA^{His} from *E. coli queA*Δ versus *tgt*Δ. (**D**) Mismatch features of tRNA^{His} from *E. coli queA*Δ and *tgt*Δ. (**E**) Comparative analysis of error rates of tRNA$^{Tyr(1)}$ from *E. coli queA*Δ versus *tgt*Δ. (**F**) Mismatch features of tRNA$^{Tyr(1)}$ from *E. coli queA*Δ and *tgt*Δ. (**G**) Comparative analysis of error rates of tRNA$^{Tyr(2)}$ from *E. coli queA*Δ versus *tgt*Δ. (**H**) Mismatch features of tRNA$^{Tyr(2)}$ from *E. coli queA*Δ and *tgt*Δ.
